# Dual Nature of Bacteriophages: Friends or Foes in Minimally Processed Food Products—A Comprehensive Review

**DOI:** 10.3390/v17060778

**Published:** 2025-05-29

**Authors:** Michał Wójcicki, Barbara Sokołowska, Andrzej Górski, Ewa Jończyk-Matysiak

**Affiliations:** 1 Bacteriophage Laboratory, Department of Phage Therapy, Hirszfeld Institute of Immunology and Experimental Therapy, Polish Academy of Sciences, Weigla 12, 53-114 Wroclaw, Poland; andrzej.gorski@hirszfeld.pl (A.G.); ewa.jonczyk-matysiak@hirszfeld.pl (E.J.-M.); 2 Department of Microbiology, Prof. Wacław Dąbrowski Institute of Agricultural and Food Biotechnology—State Research Institute, Rakowiecka 36, 02-532 Warsaw, Poland; barbara.sokolowska@ibprs.pl; 3 Phage Therapy Unit, Hirszfeld Institute of Immunology and Experimental Therapy, Polish Academy of Sciences, Weigla 12, 53-114 Wroclaw, Poland; 4 Department of Immunology, Medical University of Warsaw, Nowogrodzka 59, 02-006 Warsaw, Poland

**Keywords:** bacteriophages (phages), antibiotic resistance, biocontrol, food quality, food safety, foodborne pathogens, foodborne saprophytic bacteria, minimally processed foods

## Abstract

The increasing consumer demand for minimally processed foods (MPFs) has highlighted the need for innovative preservation methods that ensure both safety and quality. Among promising biocontrol tools, bacteriophages—viruses that selectively destroy bacteria—have gained significant attention. This review explores the dual role of bacteriophages in the food industry. On one hand, they offer a natural, highly specific, and environmentally friendly means of controlling both pathogenic and spoilage bacteria in MPFs, contributing to improved food safety, extended shelf life, and reduced reliance on antibiotics and chemical preservatives. Their use spans primary production, bio-sanitization, and biopreservation. On the other hand, phages pose significant risks in fermentation-based industries such as dairy, where they can disrupt starter cultures and impair production. This review also examines the regulatory, technological, and safety challenges involved in phage application, including concerns about antibiotic resistance gene transfer, the presence of endotoxins, and scale-up limitations. Ultimately, this paper argues that with proper strain selection and regulation, bacteriophages can become valuable allies in sustainable food systems, despite their potential drawbacks. The application of strictly virulent bacteriophages as part of “green biotechnology” could enhance food quality and improve consumer health safety. By implementing the “farm to fork” strategy, bacteriophages may contribute to the production of health-promoting and sustainable food.

## 1. Introduction

Malnutrition arises from deficiencies or poor absorption of essential nutrients, and it encompasses both undernutrition and overnutrition, including obesity—a major global cause of disease and mortality [1,2]. Diet is a key modifiable risk factor for various conditions, influencing blood pressure, glucose, cholesterol, and body weight [3,4]. Alongside poor dietary habits, smoking and inactivity significantly contribute to disease development [5,6]. Food processing also affects health outcomes [2,7]. The NOVA classification, introduced in 2009, categorizes foods based on processing level and purpose into four groups: (a) unprocessed and minimally processed foods; (b) processed culinary ingredients; (c) processed foods, and (d) ultra-processed foods [3,6,8,9]. Though endorsed by the World Health Organization (WHO), Food and Agriculture Organization (FAO), and Pan American Health Organization (PAHO), NOVA has faced criticism in the U.S. and lacks Food and Drug Administration (FDA) and United States Department of Agriculture (USDA) recognition [10,11].

Growing awareness of the link between diet and health has shifted consumer preferences toward fresh, minimally processed foods (MPFs) due to their role in preventing degenerative diseases such as heart disease and cancer [12,13,14]. However, maintaining both safety and nutritional quality in MPFs remains a challenge. Traditional methods often compromise one for the other, prompting interest in biological solutions like bacteriophages—viruses that specifically target bacteria. This review explores their dual role as tools for enhancing safety and serving as potential disruptors in MPF systems.

## 2. Minimally Processed Food

The concept of “minimal processing” generally refers to using the least amount of processing needed to achieve the desired outcome [14]. It often emphasizes preserving nutritional and sensory qualities while reducing reliance on heat [12]. Modern definitions highlight techniques that ensure shelf life and meet consumer expectations for freshness and convenience [15]. The NOVA classification offers a distinct view, defining MPFs as natural items treated with basic preservation methods (e.g., drying, cooking, freezing) without added salt, sugar, fats, or other substances. These processes aim to retain the food’s natural state, aid storage, and ensure safety. Many foods classified in the first NOVA group reflect home-cooked meals and support a healthy diet when eaten in balanced combinations [8,11,16]. Despite variations in how “minimally processed food” is interpreted, several common characteristics can be identified: (a) they retain the freshness of raw ingredients—such as texture, color, taste, and aroma—due to gentle heat treatment and preservation methods; (b) they maintain nutrients that are sensitive to processing, particularly vitamins, provitamins, phytonutrients, and minerals; (c) they often involve combined preservation methods that incorporate mild processing techniques alongside physicochemical or biological approaches; (d) packaging involves modified conditions and materials specifically tailored to the product; and (e) they require refrigeration throughout the production and distribution process to maintain quality and safety [17,18,19].

### 2.1. Microbiological Hazards in Minimally Processed Food Products

The interest in and demand for fresh, natural foods have been steadily increasing for many years, with consumers seeking low-processed products that offer high nutritional value [19,20]. This growing preference for minimally processed and functional foods presents a significant challenge for food technologists. Producing safe, MPFs with an adequate shelf life is complex and requires the development of innovative, non-thermal processing methods within the food industry [21,22,23].

MPFs can be broadly categorized into two main groups: (a) plant-based products, such as fruits and vegetables, and (b) animal-based products, including meat, fish, and seafood. In addition to these primary categories, the expanding market for low-processed foods also encompasses other product types, such as whole-grain foods, ready-to-eat meals, and items preserved using advanced techniques like cook–chill, cook–freeze, and sous-vide methods [19,24].

Minimally processed technologies often involve mechanical operations such as cutting or peeling, which can cause tissue damage and the release of cell contents at injury sites [25,26]. The leakage of cell juices during processing increases water activity in the product, creating favorable conditions for microbial growth [25,27]. Specifically, latex secreted from cut lettuce surfaces can stimulate the proliferation of enterohemorrhagic *Escherichia coli* O157:H7 (EHEC), a verotoxin-producing bacterium [28]. The elevated microbial load in MPF products often stems from the high level of microorganisms present in the raw materials. In addition to monitoring pathogenic bacteria that pose a risk to consumer health, it is equally important to address the presence of saprophytic bacteria. Their rapid growth can lead to a decline in product quality and a shortened shelf life [29,30].

### 2.2. Methods of Preserving Minimally Processed Foods

To guarantee the safety and high quality of minimally processed foods, the food industry is advancing various preservation techniques. Common methods include thermal and non-thermal processing, refrigerated storage, innovative packaging solutions (modified atmosphere packaging, controlled atmosphere packaging, edible coatings), the application of natural antimicrobials or chemicals, and the use of hurdle technology [12,17,19].

In recent years, there has been a growing shift away from physical and chemical food preservation methods in favor of biological approaches. Among the most promising biocontrol agents for extending the shelf life of fresh-cut fruits and vegetables are lactic acid bacteria (LAB) [20,31], which have demonstrated antibacterial activity against pathogens such as *Escherichia coli*, *Listeria monocytogenes*, *Salmonella* Typhimurium, and *Shigella sonnei* [32]. Both Gram-positive and Gram-negative bacteria produce bacteriocins—protein-based compounds with bactericidal or bacteriostatic properties [33,34]. Unlike antibiotics, which are produced during the dying phase of microbial growth (secondary metabolites), bacteriocins are synthesized during the stationary phase as primary metabolites [35]. These compounds target bacterial cytoplasmic membranes, disrupting proton movement, which leads to ion leakage, a loss of ATP, the release of genetic material (RNA or DNA), and ultimately cell death [36].

Another promising biological preservation method involves the use of bacterio-phages—highly specific, strictly lytic viruses that target bacteria. The application of phage-based biopreparations for food biopreservation is discussed in Section 4.4.

**Figure 1:**
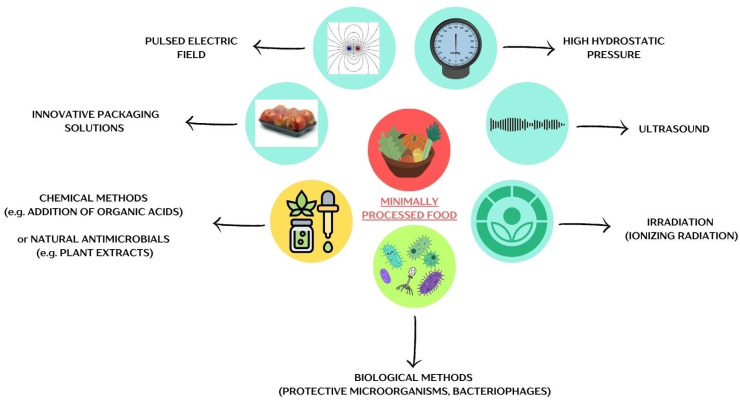
Methods of preserving minimally processed foods. The figure was prepared in Canva.

Figure 1 illustrates the primary physical, chemical, and biological methods employed in preserving MPF products.

## 3. Food as a Carrier of Antibiotic-Resistant Bacteria

**Figure 2:**
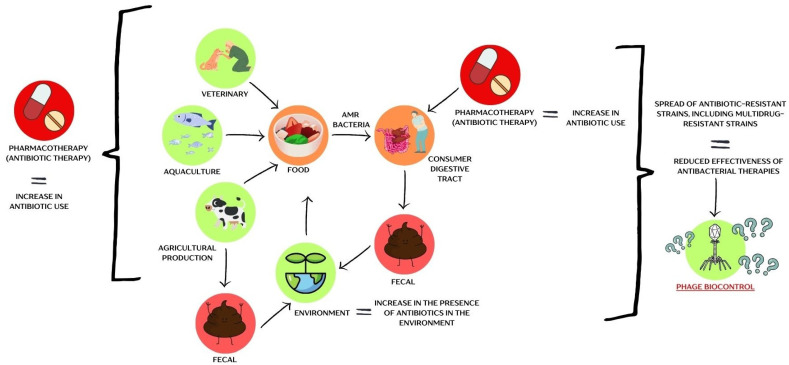
A schematic representation of the problem of antibiotic-resistant bacteria within the food chain. The figure was prepared in Canva.

The antibiotic era began in the 20th century with Alexander Fleming’s discovery of penicillin in 1928 [37,38]. The period from 1950 to 1970 is often referred to as the “golden age” of antibiotic discovery, during which half of the antibiotics we use today were identified. However, since then, no new classes of antibiotics have been discovered [39,40]. Unfortunately, poor antibiotic stewardship (AMS) has contributed to the onset of what is now known as the post-antibiotic era [41,42,43]. Antibiotics are becoming less effective in treating bacterial infections, and the rise of antibiotic resistance in microorganisms is closely linked to the presence of antibiotic resistance genes (ARGs) in the environment [44,45]. These resistance genes can be transferred to pathogenic bacteria through horizontal gene transfer (HGT) [46,47]. Conjugation, one of the primary mechanisms for spreading ARGs, bypasses the usual genetic barriers to gene transfer [44,47]. Some conjugative modules even enable resistance plasmids to be exchanged between unrelated bacterial species [44]. Numerous studies highlight that food is a significant reservoir of ARGs (Figure 2).

The rapid spread of ARGs is mainly driven by excessive antibiotic use in agriculture and HGT [44,45]. For instance, streptomycin use in the 1950s to control *Erwinia amylovora* in fruit trees led to resistance development; it remains approved in some countries outside the EU, including Israel, Canada, Mexico, and New Zealand [44]. Agricultural antibiotic use far exceeds medical use—about 80% of U.S. antibiotic sales are for farming [48]. Resistant bacteria from treated animals are excreted into the environment via feces, contaminating manure-fertilized fields and aquatic ecosystems [49]. ARGs have since been found in meat and minimally processed plant-based foods. The dairy industry also contributes to environmental ARG spread [44,47].

Inappropriate human antibiotic use—such as prescribing for viral infections or improper dosage—further worsens resistance [50,51,52]. This has led to increased prevalence of MDR strains and reduced antibiotic effectiveness [53,54]. Notably, methicillin-resistant *Staphylococcus aureus* (MRSA) causes more annual deaths in the U.S. than emphysema, Parkinson’s, or AIDS [55,56].

In response, the EU banned antibiotics as growth promoters in 2006 [57,58], and resistance now ranks among the top public health priorities in EU programs [59]. Member states must develop national action plans. In Poland, the National Antibiotic Protection Programme, now partly integrated into the National Health Programme (2021–2025), addresses this issue [60].

Eliminating all drug resistance genes is not feasible, as some have other functions unrelated to antimicrobial resistance (AMR). Therefore, efforts should focus on controlling the emergence, selection, and spread of these genes, particularly in bacteria that interact with humans, animals, and plants. Researchers are intensively working on methods to prevent HGT. Another promising approach involves combining principles from developmental biology, evolution, and ecology to combat AMR spread [44]. Increasing attention is also being given to the use of bacteriophages as a potential solution to fighting MDR strains (discussed in Section 4) [61,62,63].

### 3.1. Antibiotics and Mechanisms of Their Action

**Figure 3:**
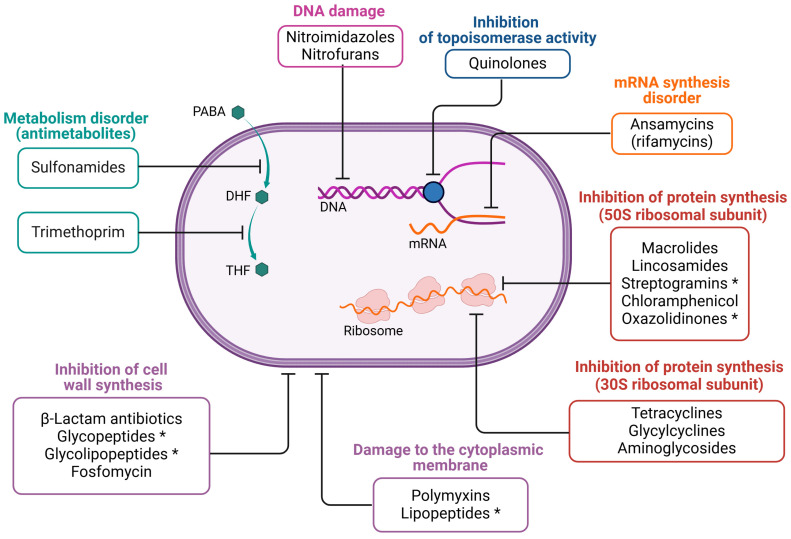
The mechanism of action of selected drug groups. The figure was created in BioRender. Wójcicki, M. (2025) https://BioRender.com/r74m836, accessed on 27 May 2025 (license no.: KO27XLU1BJ). Symbols in the figure: PABA—*p*-aminobenzoic acid; DHF—dihydrofolate; THF—tetrahydrofolate. The asterisk (*) indicates antibiotics that are active only against Gram-positive bacteria.

Antibacterial drugs are a broad group of compounds. The original definition of “antibiotics” referred to natural, secondary metabolites produced by microorganisms with antibacterial properties, while chemotherapeutics were considered synthetic chemical compounds that did not have a natural equivalent. Today, the term “antibiotics” encompasses all antibacterial drugs, including both naturally derived and synthetic chemotherapeutic agents [64,65]. Antibiotics with similar molecular structures tend to have similar efficacy, toxicity, and potential side effects [65,66]. Figure 3 illustrates the classification of antibiotics based on how they act on or within bacterial cells.

### 3.2. Antibiotic Resistance and Mechanisms of Bacterial Resistance

The development of new antibiotics has driven the emergence of drug-resistant bacterial strains. Acquired resistance arises from genomic changes, either through spontaneous mutations (e.g., DNA polymerase errors) or via transposable elements. HGT also facilitates resistance spread through conjugation, transformation, and transduction. Key contributors include mobile genetic elements (e.g., plasmids, phages, integrons), recombination processes, and selective pressure [64]. Definitions of acquired antibiotic resistance were established by experts from the European Centre for Disease Prevention and Control (ECDC) in Stockholm and the US Centers for Disease Control and Prevention (CDC):

i. MDR (multidrug resistance): the strain shows resistance to at least one antibiotic from three or more different classes of antibacterial drugs;
ii. XDR (extensively drug resistance): the strain is resistant to at least one antibiotic from all but a maximum of two classes of antibiotics used to treat infections caused by the microorganism;
iii. PDR (pandrug resistance): the strain is resistant to all available, approved antibiotics from all categories used to treat infections caused by the specific microorganism species [67,68,69,70].

Over time, bacteria have evolved various mechanisms to resist antibiotics, including (a) target site alteration; (b) reduced membrane permeability; (c) drug-inactivating enzymes; (d) efflux pumps; (e) alternative pathways or enzymes; and (f) protective proteins shielding drug targets [70,71].

### 3.3. Natural Resistance of Bacteria to Antibiotics

In addition to acquired resistance, bacteria also exhibit natural resistance, which is commonly observed within a particular family, genus, or species. This type of resistance is not linked to prior antibiotic exposure and does not involve HGT. Natural resistance arises from (a) the absence of a target site for the drug within the bacterial cell, (b) the presence of structures that block the drug from reaching its target, such as lipopolysaccharides in the outer membrane of Gram-negative bacteria, or (c) the activity of specific resistance mechanisms, where effectors like enzymes or membrane transporters are encoded by chromosomal genes characteristic of the bacterial species [70,72,73].

## 4. Bacteriophages as a Natural Method of Biocontrol of Bacterial Microbiota in Food

The growing prevalence of MDR strains and the increasing frequency of infections caused by these bacteria have prompted the search for alternative antibacterial agents to traditional antibiotics [74,75]. One potential solution is the use of bacteriophages—natural enemies of bacteria. Phage therapy is not a new concept, as it was practiced even before the discovery of antibiotics [76,77,78]. For many years, experimental, targeted phage therapy has been carried out in Poland at the Phage Therapy Unit of the Medical Center of the Ludwik Hirszfeld Institute of Immunology and Experimental Therapy of the Polish Academy of Sciences (HIIET PAS) in Wroclaw [79,80,81].

The rising demand for minimally processed food, where maintaining structure and nutritional value is essential, has led the food industry to explore new, non-invasive preservation methods [82,83]. The use of bacteriophages throughout the food production chain—such as directly eliminating bacteria from food surfaces and processing equipment in contact with the product—could help improve the microbiological safety of minimally processed food [84,85,86,87]. Since bacteriophages are obligate bacterial parasites, they naturally coexist with the food microbiota and could serve as natural biological agents targeting their bacterial hosts [88,89,90].

### 4.1. Phage Particle Structure

Bacteriophages (phages), like eukaryotic viruses, lack the ability to amplify independently and do not have a functional metabolism or cellular structure [91,92]. Despite this, phages undergo evolutionary processes and exhibit variability. Their genome is composed of nucleic acids, which may include configurations not commonly found in cellular genomes, such as double-stranded RNA (dsRNA) [91,93]. The unique characteristics of phages place them at the intersection of living and non-living matter [92]. Virus classification typically relies on analyzing virion morphology through transmission electron microscopy (TEM) and genome sequencing methods [94,95]. The International Committee on Taxonomy of Viruses (ICTV) is responsible for the taxonomic classification of viruses, including bacteriophages [94,96]. However, there are no universal criteria for defining the genus or species of individual viruses. The ICTV adopts a polythetic species concept, where a species is identified by a set of common features, some of which may be absent in certain members of the species [97]. According to ICTV guidelines, subfamilies are only created when they provide additional hierarchical information [98]. A genus is defined as a group of viruses with over 70% nucleotide sequence similarity, distinct from other viral types, while a species is a monophyletic group of viruses distinguishable by multiple criteria. A species is the lowest taxonomic level recognized by the ICTV [99].

The primary demarcation criterium for defining bacteriophage species is a genome sequence similarity of at least 95%, meaning that two viruses within the same species can differ by no more than 5% in nucleotide sequence alignment [98]. In 2020, the ICTV adopted a binomial naming system for virus species, where the genus name and species epithet together create a unique species name [100,101]. The Bacterial Viruses Subcommittee (BVS) implemented a fully open binomial format, allowing research groups and authors of taxonomic proposals to determine the final species name format [102]. Classifying bacteriophages based on experimental data is both time-consuming and labor-intensive, which has led to the exploration of machine learning as an appealing alternative [103,104,105]. New tools for supporting bacteriophage classification include applications like INPHARED, GRAViTy, vConTact2, virClust, PhaGCN2, and PhageAI [106,107,108,109,110].

Phage particles are composed of genetic material in the form of either single-stranded or double-stranded DNA or RNA, encased in structural proteins that form a capsid. The capsid can have a helical (filamentous or rod-like; such as phage M13), isometric (icosahedral or spherical, such as phage ΦX174), or icosahedral shape, often connected to a tail, forming a complex (tailed) structure [111,112,113]. The phage genome contains all the necessary information for replication within the bacterial host cell, the synthesis of phage proteins, and the assembly of progeny phages capable of infecting other bacterial cells [114]. The protein capsid is highly resistant to most external environmental factors, safeguarding the genetic material inside [111,115]. Many phage nucleocapsids are surrounded by an additional lipid membrane, which is a fragment of the host cytoplasmic membrane, forming an envelope connected to the capsid by matrix proteins. In contrast, non-enveloped (naked) viruses lack this lipid membrane [116,117].

Typical examples of phages with complex structures are virions belonging to the *Caudoviricetes* class, which includes both bacterial and archaeal viruses containing a double-stranded DNA (dsDNA) genome [118,119,120]. In 2022, the BVS recognized the *Caudoviricetes* class, removing the *Caudovirales* order, including previous morphological *Myoviridae*, *Podoviridae*, and *Siphoviridae* families, and introducing a binomial system for species naming [102]. Research on the classification of newly discovered bacteriophage sequences is ongoing, and as of 30 April 2025, the *Caudoviricetes* class includes 11 orders, 105 families, 132 subfamilies, 1680 genera, and 5799 species of bacterial and archaeal viruses [121].

### 4.2. Bacteriophage Replication Cycles

The first step in phage infection is the adsorption of virions to the surface of the bacterial host cell [122,123]. This process varies depending on the type of bacteria. In Gram-negative bacteria, phage attachment occurs in two stages. Initially, the phage binds reversibly to lipopolysaccharides (LPSs) that contain the polymannose O antigen, and then it attaches permanently to the ferrichrome transporter [124,125,126]. Ferrichromes are siderophores, compounds that bacteria secrete to capture iron ions from the environment [127]. For Gram-positive bacteria, phage attachment involves the binding of the phage to specific sugar receptors in the bacterial cell wall. These receptors may include glucose, galactose, rhamnose, glucosamine, or galactosamine [124,128]. In some cases, the interaction between the phage and bacterial host may require the involvement of bacterial phage infection proteins (PIPs) or membrane proteins [129,130].

**Figure 4:**
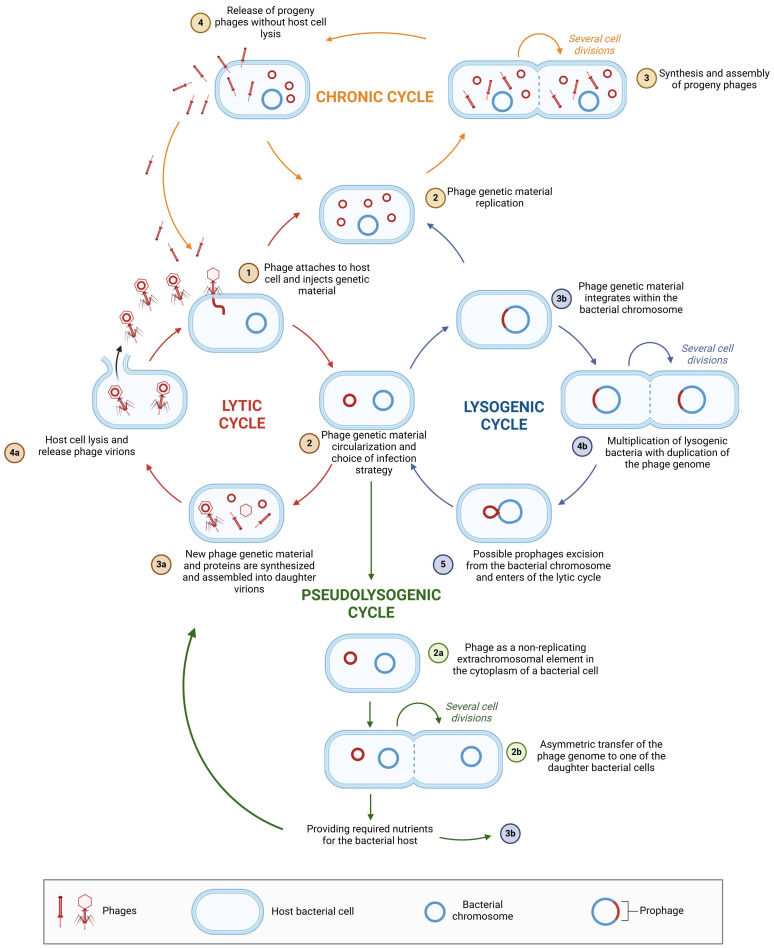
Possible phage–bacterial host interactions. The figure was created in BioRender. Wójcicki, M. (2025) https://BioRender.com/r74m836, accessed on 27 May 2025 (license number: FR27XLSFWZ).

Environmental factors, as well as the physiological and genetic traits of the bacterial host, can lead to variations in phage replication pathways [129]. Phage replication strategies exist on a spectrum, from highly productive infections that result in the release of new virions to persistent nonproductive infections that do not generate new phages but instead spread prophage genomes within the bacterial population (transferred from parent to daughter cells) [131,132]. The two most well-known and studied phage replication pathways are the lytic and lysogenic cycles [95,133]. A variation of the lysogenic cycle is the carrier state, also known as the pseudolysogenic cycle [124]. Another form of phage–host interaction is chronic infection [124,131]. Figure 4 illustrates the differences between these various phage infection strategies.

### 4.3. Benefits and Risks of Using Bacteriophages

Phage therapy refers to the use of phages and/or their enzymes to treat bacterial infections. This approach offers several advantages over antibiotic therapy [124,134]. However, despite its benefits, phage application also carries certain risks that must be considered when selecting phages for the development of effective biocontrol preparations.

Bacteriophages, as viruses that infect prokaryotic cells, can replicate only within bacterial hosts, making them harmless to human, animal, and other eukaryotic cells [124,135]. They are a natural part of the human gut microbiome [92,136,137] and have also been found in blood, the genitourinary tract, the respiratory system, the skin, and cerebrospinal fluid [138,139]. The gut microbiota is a complex ecosystem containing trillions of microbial cells, including bacteria, archaea, eukaryotes, and viruses. It plays a crucial role in human health, contributing to the biosynthesis of vitamins, amino acids, and short-chain fatty acids (SCFAs), supporting immune system development, and providing protection against pathogens [140,141]. Disruptions in the gut microbiome have been linked to various diseases, such as inflammatory bowel disease, obesity, diabetes, and neurological disorders [142,143]. Bacteriophages are the most abundant component of the gut microbiota, with an estimated 10^15^ phage particles in the human gastrointestinal tract. They significantly influence the composition, function, and modulation of the gut microbiome [144,145]. Each individual has a unique “phageome”, with only about 5% of phages forming a core common to all humans [146]. The structure and function of the gut phage community are shaped by multiple factors, including environmental influences, gut microbiota composition, and host immune responses [92,146,147,148]. Phages and bacteria exhibit a distinct distribution within the intestines. Temperate phages and numerous bacteria are found closer to the intestinal lumen, while deeper layers of the mucosa contain lytic phages and fewer bacteria resistant to phage infection [92,147,148]. Despite their high morphological, structural, and genetic diversity, intestinal phages share certain characteristics. Most are non-enveloped and possess DNA genomes, though some RNA phages are present, likely originating from ingested plant-based food [136,149]. A promising emerging approach involves using lytic phages as probiotics or dietary supplements to target and eliminate harmful bacteria. The key distinction between bacterial probiotics and lytic phage-based probiotics, known as phagobiotics, lies in their mechanisms of action. While bacterial probiotics utilize nonpathogenic bacteria to inhibit the colonization and pathogenicity of harmful bacteria, phagobiotics employ lytic phages to selectively eliminate specific pathogens while preserving the beneficial microbial community. Due to their high specificity, phagobiotics are expected to have minimal impact on the overall microbiota and may even act synergistically with traditional probiotics [150]. Numerous studies have explored potential interactions between bacteriophages and eukaryotic cells, demonstrating their influence on biochemical and physiological processes [139,151]. Although phages do not trigger acute immune responses, they can affect tissue and organ function. Research has shown that phages can enter breast epithelial cells via endocytosis and reach the nucleus, suggesting a role in transcytosis across epithelial barriers [136,139]. Additionally, phages have been found to modulate gene expression in eukaryotic cells [139,152]. The therapeutic effects of phage preparations may extend beyond their bactericidal activity, as they can also stimulate immune system cells and enhance proliferation in response to mitogens [153]. Phage lysates, which contain bacterial remnants following lysis—such as endotoxins—can also influence immune responses [152,154]. Studies indicate that phages may exhibit immunomodulatory properties by affecting phagocyte functions, including phagocytosis, reactive oxygen species (ROS) production, and T lymphocyte proliferation [139,151,155]. Additionally, they possess immunogenic properties, stimulating the production of specific antibodies against phage antigens. Bacteriophages interact with innate immune cells, inducing phagocytosis and influencing antimicrobial protein levels, as well as modulating cytokine production by other immune cells. Due to their nucleoprotein structure, phages are recognized by immune system cells, leading to their neutralization and clearance from the body [156]. Furthermore, they can influence the adaptive immune response by stimulating the production of antiphage antibodies [136,139].

One of the key advantages of bacteriophages is their ability to increase their dose automatically. As self-amplifying entities, phages replicate in the presence of their bacterial hosts, accumulating precisely where they are needed [157,158]. A significant benefit of phage therapy is its effectiveness against multidrug-resistant bacteria, helping to control infections caused by resistant strains [158,159]. Additionally, bacteriophages through enzymes encoded in their genomes can penetrate and disrupt bacterial biofilms, improving access for other antibacterial agents [160,161,162]. Phages are highly specific to their bacterial targets, which can be both an advantage and a limitation. Their precise targeting makes them valuable for therapeutic applications and biocontrol in the agri-food industry. However, to ensure broad efficacy, phage-based treatments should consist of cocktails containing multiple phages with a polyvalent specificity [163,164]. On the other hand, this strain specificity prevents phages from harming beneficial bacteria used in biotechnological and food industries, such as dairy starter cultures [165,166]. Using phage cocktails or polyvalent phages (capable of infecting multiple bacterial strains) may introduce complex pharmacological interactions. In some cases, these interactions can lead to antagonistic effects, reducing phage efficacy and resulting in bacteriostatic rather than bactericidal activity [163].

For both therapeutic applications and the development of biopreparations for the agri-food industry and bioremediation, only virulent phages should be identified and selected [167,168]. This is because temperate phages, which integrate into the bacterial genome during the lysogenic cycle, can facilitate HGT, including the spread of antibiotic resistance genes [155,169,170,171]. Prophages often encode virulence factors within pathogenicity islands (PAIs), a subset of genome islands (GEIs), and lysogenic conversion can contribute to the pathogenicity of certain bacteria [172,173]. A notable example is *Vibrio cholerae*, where virulence is driven by transducing phages CTXΦ and VPIΦ, known as choleraphages, which carry virulence-related genetic elements in their genomes [174,175]. The virulence cassette within these phages contains four key genes that contribute to bacterial pathogenicity: (a) *ctxAB*—encodes cholera toxin (CT), a cytotoxic AB-type enterotoxin. Its A subunit induces intracellular cAMP (cyclic adenosine monophosphate) production, disrupting ion transport and causing severe fluid loss; (b) *zot*—encodes the zonula occludens toxin (ZOT), which disrupts intercellular junctions, increasing intestinal permeability; (c) *ace*—encodes the accessory cholera enterotoxin (ACE), which contributes to fluid accumulation in the intestine; and (d) *cep*—encodes fimbriae, aiding bacterial colonization of the small intestine [174,176,177].

Over the course of evolution, bacteria have developed various defense mechanisms against bacteriophage infections, acting at different stages of the infection process [178,179]. One such mechanism is adsorption inhibition, which prevents phage attachment by physically masking receptors, altering their structure, or eliminating them entirely. The masking process often involves the synthesis of extracellular polysaccharides, which form an additional protective layer that blocks phage recognition sites. Additionally, spontaneous mutations in bacterial genes responsible for receptor synthesis or transport can lead to the loss or malfunction of these receptors, making bacterial cells resistant to phage adsorption [179,180]. Other bacterial defense strategies include blocking phage DNA injection through the superinfection exclusion (Sie) system, which prevents foreign phage genomes from entering the cytoplasm [180]. Another important mechanism is abortive infection (Abi), which operates within the cytoplasm to disrupt various stages of phage replication, ultimately leading to the programmed cell death (apoptosis) of infected bacteria. This self-sacrificial strategy prevents the spread of phages within the bacterial population, ensuring the survival of uninfected cells [180,181,182,183,184]. Additionally, phages themselves can encode membrane-bound Sie proteins which protect against the entry of genetic material from competing lytic phages [182]. The restriction–modification (R-M) system is another bacterial defense mechanism that involves the recognition and destruction of foreign DNA through endonucleolytic digestion. Bacterial DNA is protected from degradation by specific methylation modifications introduced by enzymes with methyltransferase activity [183,185,186]. A more advanced bacterial immune defense mechanism is the CRISPR-Cas system (clustered regularly interspaced short palindromic repeats), which enables bacteria to recognize and degrade foreign genetic material. This system consists of short DNA fragments (spacers) homologous to phage genomes, separated by palindromic sequences within the bacterial genome. The associated Cas proteins degrade the invading phage DNA through a three-stage process: (1) adaptation—the bacterium incorporates a new spacer sequence from the phage genome into its CRISPR array; (2) expression—the CRISPR array is transcribed into CRISPR RNA (crRNA), which contains a single spacer sequence; and (3) interference—the crRNA associates with effector proteins that scan invading DNA for complementarity. If a match is found, the phage genome is degraded by Cas nucleases, preventing bacterial lysis [179,187,188,189,190]. Other bacterial defense systems include the DISARM (defense island system associated with restriction–modification), which consists of five genes encoding a DNA methyltransferase, DrmA helicase, DrmB (a protein of unknown function), phospholipase D domain nuclease (DrmC), and an accessory protein. This system functions by methylating bacterial DNA while restricting and modifying invading phage genomes [181,191,192]. Similarly, the BREX (bacteriophage exclusion) system prevents phage replication and integration through bacterial DNA methylation [193,194]. Recent discoveries have revealed additional bacterial antiphage defense systems, though their exact molecular mechanisms remain unknown and require further research [179,195,196].

Bacteriophages and bacteria are engaged in a continuous evolutionary arms race, each adapting to outcompete the other for survival. Phages evolve to overcome bacterial defense mechanisms by recognizing and developing alternatives to altered bacterial receptors, allowing them to effectively adhere to host cells [182]. When bacteria modify their receptors, phages counteract by producing polysaccharide-degrading enzymes, such as lyases and hydrolases, to break through bacterial defenses [197]. Phages can also bypass bacterial modification-dependent systems and deploy their own anti-CRISPR mechanisms to evade acquired bacterial resistance [182]. Additionally, they can escape CRISPR-Cas defenses through mutations in protospacer-adjacent motif (PAM) sequences, preventing recognition and cleavage by bacterial nucleases [198]. Some phages have evolved protective genomic modifications, such as glucosylated hydroxymethylcytosine (HMC) or methylated DNA, which shield them from bacterial restriction enzymes. For example, Staphylococcus phage K can eliminate Sau3A restriction sites on both DNA strands to evade degradation [199,200]. Similarly, bacteriophage T4 protects itself by glucosylating HMC residues, making its genome resistant to bacterial restriction–modification systems [201]. Phages can also produce proteins that inhibit bacterial defenses. For instance, some phages synthesize a 76-amino-acid restriction nuclease inhibitor (IPI*), which binds to bacterial GmrS and GmrD proteins, preventing phage DNA digestion [202]. Mutations in nucleotide metabolism genes allow phages to escape the Abi system, particularly the AbiQ mechanism [182,203]. To counteract bacterial toxin–antitoxin (TA) systems, bacteriophage ΦTE produces pseudoantitoxin RNA (pseudo-ToxI), which mimics bacterial antitoxins and neutralizes apoptosis-associated defense responses [204].

### 4.4. Invisible Guardians: The Role of Bacteriophages in Food Safety

Phages can be applied across three key sectors of the agri-food industry: (a) primary production, including animal farming and crop cultivation; (b) bio-sanitization, primarily in manufacturing facilities to prevent biofilm formation on equipment surfaces; and (c) biopreservation, which focuses on extending the microbiological shelf life of food by inhibiting the growth of both pathogenic and spoilage bacteria [83,205].

Given the potential risks associated with bacteriophage applications, phage preparations should comply with strict safety criteria: (a) they should be classified as organisms generally recognized as safe (GRAS); (b) they must be strictly lytic (virulent); (c) for pathogenic bacterial biocontrol, they should be capable of replicating in nonpathogenic host cells; and (d) their genomes must be free of toxin- and antibiotic resistance-related genes [206,207]. Additionally, bacteriophages used in biopreparations should exhibit a broad bacterial host range, high stability under various storage conditions (resistance to pH fluctuations, temperature changes, and gastrointestinal enzymes), and the ability to replicate efficiently, producing high phage titers [208,209,210,211].

#### 4.4.1. Primary Production

Bacterial phytopathogens significantly reduce crop yields and thus pose a threat to food supply [212]. The use of bacteriophages is considered a potential biocontrol measure in sustainable primary production. Phage preparations specifically targeting major bacterial plant pathogens (including *Xanthomonas*, *Pseudomonas*, *Erwinia*, or *Pectobacterium*) in field cultivation could be applied in the form of sprays or surface treatments [213,214]. Unlike pesticides, phages as biodegradable particles do not leave harmful chemical residues in the environment [215,216]. Compared to antibiotics or copper-based sprays, they also exert lower selective pressure for resistance [213]. Phages have already been tested or applied to combat diseases such as bacterial fruit blotch, goss wilt, soft rot and blackleg, fire blight, bacterial canker, bacterial leaf blight, or pepper bacterial spot [212]. However, regulatory hurdles and the need for cost-effective large-scale production still limit their widespread adoption. Despite these challenges, some commercial phage products, such as AgriPhage in the USA, have been registered as biopesticides by the U.S. Environmental Protection Agency and are being marketed. AgriPhage phage cocktails combat bacterial spot and speck disease in tomatoes and peppers (“AgriPhage”), tomato bacterial canker (“AgriPhage-CMM”), fire blight in apple and pear (“AgriPhage-Fire blight”), and citrus canker (“Agriphage-Citrus canker”) [212,217]. Another preparation available in the US is “Xylphi-PD”, which targets Pierce’s disease of grape (related to *Xylella fastidiosa* infection) [217]. In Europe, only a very limited number of commercial phage bioproducts are available. The Scottish company APS Biocontrol sells the post-harvest food processing aid “BioLyse-PB”, which prevents *Pectobacterium* infections in potato tubers, while the Hungarian company Enviroinvest has received authorization for local sales of a phage cocktail targeting *E. amylovora* (“Erwiphage Plus”) [212,217]. However, to date, the EFSA has not registered any phage-based product as a plant protection agent or biopesticide.

Phages may also find application in targeted approaches to controlling bacterial infections such as *Salmonella*, *E. coli*, and *Clostridium* in livestock. By reducing the bacterial load and preventing disease outbreaks, phages can help improve survival rates, growth performance, and feed efficiency, which are key in breeding programs [218,219,220,221]. In addition, phages can selectively modulate the gut microbiome, promoting beneficial bacteria that enhance nutrient absorption and immune function, thereby indirectly supporting better reproductive outcomes. An important advantage of using phages is the reduction in antibiotic use, which helps lower the risk of developing antibiotic-resistant bacteria—a major global issue in both human and veterinary medicine. Phages may also play a role in improving reproductive health by treating infections in the reproductive system, potentially increasing fertility rates in breeding animals [222,223]. Current research focuses on developing phage-based feed additives, sprays, and modified phages with a broader host range or enhanced activity, as well as integrating phages into comprehensive herd health management strategies [224,225]. The Polish biotechnology company Proteon Pharmaceuticals specializes in developing commercial bacteriophage preparations aimed at improving animal health, reducing antibiotic use, and supporting sustainable livestock farming and aquaculture. It offers feed additives for poultry targeted at combating *Salmonella* (BAFASAL^®^ and BAFASAL^®^ + G) and avian pathogenic *E. coli* (BAFACOL^®^). Additionally, it produces products for aquaculture. The BAFADOR^®^ preparation, targeting *Aeromonas* and *Pseudomonas*, aims to maintain gut microbiota balance, support fish welfare, and increase the economic profitability of farming. Proteon Pharmaceuticals’ products are commercially available in selected markets in Southeast Asia, South America, and Africa [226,227,228,229,230].

#### 4.4.2. Bio-Sanitization

Phages are becoming innovative tools in the bio-sanitation of food production lines. Unlike traditional chemical disinfectants, phages are highly specific, targeting only host bacteria without disrupting beneficial microbial communities or leaving chemical residues on equipment or surfaces [231,232]. This makes them particularly valuable when used against persistent bacterial pathogens such as *L. monocytogenes*, *Salmonella* spp., and *E. coli*, which can form biofilms on production line surfaces and resist conventional cleaning methods [232,233,234,235]. Phage-based bio-sanitation involves directly applying phage preparations onto food contact surfaces and production equipment [219,236,237]. These preparations can penetrate biofilms and effectively reduce bacterial loads, enhancing food safety while complying with regulatory and environmental standards. Moreover, phages are generally considered safe, making them a viable addition to integrated sanitation protocols in the food industry [232,237]. This approach enhances food safety, supports clean-label requirements, and may help combat antimicrobial resistance. Ongoing research aims to optimize phage application methods, formulations, and combinations with other sanitation strategies to improve efficacy and minimize the risk of bacterial resistance. As demand grows for safer, more sustainable food production, phage bio-sanitation stands out as an innovative tool for maintaining hygiene and ensuring the microbiological safety of food products [156,238,239].

#### 4.4.3. Biopreservation

In the food industry, commercial phage preparations have emerged as promising biocontrol tools to enhance food safety by targeting foodborne bacterial pathogens such as *L. monocytogenes*, *Salmonella* spp., *E. coli* O157:H7, and *Campylobacter* spp. [225,240,241]. Information on commercially available phage preparations for food biocontrol and legal aspects is presented in Section 4.7.

Research institutions worldwide are exploring the potential of phage biopreparations as a means of preserving MPF products. Recent selected studies on the preservation of plant-based MPF products conducted over the past five years are summarized in Table 1.

### 4.5. The Dark Side of Phages: Challenges in Food Production and Fermentation

Bacteriophages may find applications in many branches of the agri-food industry. However, in the dairy industry, bacteriophages pose a serious problem because they can cause lysis of the starter culture, leading to slowed fermentation or complete inhibition of microbial growth [205,254,255]. This, in turn, leads to a decrease in the quality of the final product or its spoilage [165,256]. It is estimated that about 70% of technological process disturbances in the dairy industry (in the production of curd and rennet cheeses) are related to phage infections. The biotechnological processes carried out in the plant are susceptible to infections because the raw material is not sterile. The use of the same starter culture (bacterial inoculum) further supplies host cells for phage development [257]. The primary sources of bacteriophages in the dairy industry are raw milk and starter cultures, while secondary contamination can occur via inadequate hygiene practices by employees (e.g., contaminated clothing) or production and ventilation equipment. Many phages retain their activity during pasteurization and spray drying processes. Starter cultures, particularly in the case of *Lactococcus* spp. strains, may in turn contain prophages capable of induction. Especially sensitive is *Lactococcus lactis* subsp. *lactis* var. *diacetylactis*, which is responsible for shaping the sensory characteristics of many dairy products [256]. Raw milk delivered to dairies may contain up to 10^4^ PFU mL^−1^ of bacteriophages. The phage titer in whey can reach up to 10^9^ PFU mL^−1^, making it the main secondary source of bacteriophages in the dairy plant. Aerosols generated from whey can lead to air contamination at levels up to 10^8^ PFU m^−3^ [258].

Phage detection in dairy production relies on monitoring acidification and performing lysogeny tests. In addition, qPCR or PCR methods (with a detection limit of 10^3^ PFU mL^−1^) are employed, as well as flow cytometry, which requires prior removal of lipid droplets from the samples [205,254]. Compliance with hygiene standards in production, the use of starter culture rotation, and the acquisition of phage-resistant strains reduce the risk of phage infections. Modern phage infection control methods include the use of phage-resistant starter culture strains free of prophages, the use of lyophilized or frozen concentrated starter cultures (DVI/DVS), and early addition of rennet, which, in combination with elevated temperature, limits the absorption of certain phages. The possibility of controlled selection of phage-resistant strains may be provided by the use of the CRISPR/Cas system [259]. Media that inhibit phage replication and aseptic inoculation systems are also used, along with separation of the CIP (clean-in-place) systems between starter culture preparation and production areas, and the use of HEPA filters. Destruction of phages with chemical agents can involve treatment with 0.05% sodium hypochlorite or potassium permanganate solutions for 5 min at room temperature [165].

### 4.6. Risks and Implications of Contaminants in Phage-Based Preparations

The application of bacteriophages in the food industry has gained increasing interest due to their specificity and effectiveness in controlling foodborne pathogens. However, the production and use of phage-based preparations are associated with several safety concerns, primarily related to the presence of contaminants originating from the bacterial host or the culture medium [260,261,262,263,264]. One of the most critical risks involves endotoxins, particularly LPS, which are components of the outer membrane of Gram-negative bacteria frequently used as host strains in phage amplification. These endotoxins are known for their pyrogenic and immunostimulatory properties and can pose significant health risks if not properly removed, even when phages are applied externally to food products [261,262].

In addition to endotoxins, there is a risk of contamination with exotoxins, which are toxic proteins secreted by certain bacterial hosts during growth. These substances, if retained in crude lysates, may contribute to toxicity or allergenicity. Furthermore, bacterial cell debris, host DNA, enzymes, and residual proteins may persist through insufficient purification processes and negatively impact the safety or regulatory compliance of the final product [262,263,264]. Residues from nutrient-rich culture media, such as peptones or yeast extract, may also remain in the preparation, potentially affecting product stability or triggering unwanted reactions. Another concern is the inadvertent inclusion of non-target or temperate bacteriophages, which could facilitate HGT or unpredictably disrupt microbial communities [260,263].

Scaling up bacteriophage production for food industry use also presents significant technological challenges [265,266]. In upstream processes, the selection of suitable host strains is crucial; these strains must be nonpathogenic, genetically stable, and incapable of producing harmful byproducts. Controlled bacterial lysis is essential to ensure efficient phage release while minimizing the liberation of cellular contaminants. Downstream processing, which includes purification, concentration, and formulation, is often complex and costly. Techniques such as ultrafiltration, chromatography, and ultracentrifugation are employed to reduce endotoxin levels and remove debris, but these methods can reduce phage viability and overall yield [260,267,268].

Concentration and stabilization of phage preparations to reach high titers (typically 10^9^–10^11^ PFU mL^−1^) without compromising their infectivity is another critical hurdle [260]. The formulation must also ensure long-term stability under various environmental conditions, particularly for industrial food applications where shelf life and robustness are key. In addition, the absence of standardized production protocols leads to variability in product quality, making regulatory approval and batch-to-batch consistency more difficult to achieve [269].

These limitations have important implications for the food industry. Contaminants, even in trace amounts, may affect regulatory acceptance by bodies such as the FDA or EFSA and could result in delays, recalls, or loss of consumer trust. Moreover, the high costs of advanced purification technologies and the need for rigorous quality control present economic barriers to large-scale implementation.

In summary, while bacteriophages offer a promising and natural alternative for pathogen control in food systems, their safe and effective use requires overcoming substantial technological and regulatory challenges. Addressing contamination risks and optimizing large-scale production processes will be essential steps toward integrating phage-based solutions into mainstream food safety practices.

### 4.7. Legal Aspects Related to the Use of Bacteriophages in the Agri-Food Industry

Currently, only a few centers in the EU use bacteriophages for therapeutic purposes, the leading ones being the Phage Therapy Unit of the Medical Centre of the HIIET PAS in Wroclaw [73,270,271] and the Queen Astrid Military Hospital in Brussels [272,273]. Despite extensive research on bacteriophage biology and their effects on the human immune system, phage therapy in Poland remains an experimental approach, with no government funding to support its clinical use [270,274]. In the agri-food sector, the use of phages as antibacterial agents is also highly restricted. While there have been successful applications of bacteriophages in food biocontrol, their incorporation into food products is only allowed in certain countries, with regulations applying exclusively to specific commercial phage-based products [275]. Within the EU, phage preparations have not yet been approved for direct contact with food [205,276,277]. However, several non-EU countries, including the United States, Brazil, Israel, Canada, Switzerland, Australia, and New Zealand, permit the use of phages in the food industry. Commercial biotechnology companies offer phage-based biopreparations targeted at food safety, with many of these products receiving FDA approval. Notable FDA-approved phage preparations include Listex^TM^ P100, Secure Shield E1, EcoShield^TM^, ListShield^TM^, ShigaShield^TM^, and SalmoFresh^TM^, and USDA-approved products include Ecolicide^®^, SalmoFresh^TM^, and Finalyse^®^ [278,279,280]. Ten commercial phage preparations have earned GRAS status from the FDA, ensuring that their endotoxin content is below 250 endotoxin units per milliliter [281]. For phage therapy solutions administered intravenously, the permissible endotoxin level is set at 5 EU per kilogram of body weight per hour [282,283]. Given the empirical nature of phage dosing, further research is necessary to fully understand the pharmacokinetics of phage biopreparations [282]. Some commercial phage biopreparations are Kosher- and Halal-certified and are approved for use in organic food production [278]. The Polish company Proteon Pharmaceuticals S.A. (Łódź, Poland) also develops and markets phage biopreparations for use as feed additives in animal husbandry [79]. One of their products, Bafasal^®^, containing four strictly lytic bacteriophages specific to *S. enterica* serovar Gallinarum strain B/00111, has received a positive opinion from the Panel on Additives and Products or Substances used in Animal Feed (FEEDAP), at the request of the European Commission. Bafasal^®^ is designed to be used as a zootechnical additive in drinking water and complementary liquid feed for all bird species [284,285].

In 2016, the European Food Safety Authority (EFSA) published a report [286] confirming the toxicological safety and efficacy of Listex™ P100 by Micreos Food Safety, while recommending further studies to assess the preparation’s effectiveness. In the United States, when Listex™ P100 (targeting *L. monocytogenes*) is used on ready-to-eat products, meat, and poultry, the manufacturer is not required to include information about the preparation on the food label, provided permissible doses are followed. The approved limit is up to 10^9^ PFU g^−1^, with a maximum of 50 ppm of potassium lactate (as Listex™ P100 contains potassium lactate) [287,288]. While Listex™ P100 is a single-phage preparation (utilizing phage P100), another commercially available biopreparation for eliminating *L. monocytogenes* is ListShield™ produced by Intralytix, Inc. This product contains six bacteriophages: LIST–36, LMSP–25, LMTA–34, LMTA–57, LMTA–94, and LMTA–148. ListShield™ can be used as a food biopreservative and for food processing in products like fish and shellfish, fresh and processed fruits, vegetables, and dairy products at a concentration of 10^6^ PFU g^−1^ [275,289].

In October 2023, the European Medicines Agency (EMA) published the “Guideline on quality, safety and efficacy of veterinary medicinal products specifically designed for phage therapy” [290], while in August 2024, the EFSA issued the “EFSA statement on the requirements for whole genome sequence analysis of microorganisms intentionally used in the food chain” [291], which opens up the possibility of using phage biopreparations in the agri-food industry in the future.

## 5. Conclusions

Bacteriophages play a dual role in the food industry—acting as both beneficial agents and potential threats. On the positive side, their usefulness is most evident in ensuring food safety, especially for minimally processed plant- and animal-based products. Phages applied in biocontrol, bio-sanitation, and biopreservation effectively combat pathogenic and spoilage bacteria, helping to extend shelf life and reduce the need for chemical preservatives. Moreover, thanks to their high specificity, they do not harm beneficial microorganisms and can target antibiotic-resistant strains, making them a valuable tool in the fight against antimicrobial resistance.

However, phages also have a dark side. In fermentation industries, particularly in dairy production, their presence poses a serious threat. By infecting starter cultures, phages can slow down or completely halt fermentation processes, resulting in reduced product quality or spoilage. Many dairy plants must invest in costly measures to limit the risk of phage contamination in production lines. Additionally, not all phages are suitable for industrial applications—temperate phages carry the risk of transferring antibiotic resistance or virulence genes, so only strictly lytic phages, which lead to complete bacterial cell lysis, should be used for biocontrol purposes.

In summary, bacteriophages can be both friends and foes of the food industry. With proper selection, quality control, and advanced purification technologies, their potential as natural, effective biocontrol agents clearly outweighs the risks. As a result, they may become an important part of strategies to enhance food safety, reduce chemical use, and address the global challenge of antibiotic resistance.

## Figures and Tables

**Table 1:** Biocontrol of saprophytic and pathogenic bacteria in minimally processed plant-based food products based on single phages and/or their cocktails.

Saprophytic Bacteria				
Food Matrices (Storage Conditions)	Bacteria	Phage/Phage Cocktail (Application Method)	Main Results	Reference
alfalfa sprouts kale sprouts lentil sprouts sunflower sprouts radish sprouts (20 °C/350 mbar)	natural bacterial microbiota of the product	cocktail of 18 phages (spraying and an absorption pad)	The spraying method was significantly more effective, achieving a maximum reduction of 1.5 log CFU g^−1^ after 48 h, while phage-soaked pads reduced bacterial counts by only 0.27–0.79 log CFU g^−1^.	[77]
rucola (20 °C/protective atmosphere)	natural bacterial microbiota of the product	cocktail of 43 phages (spraying, and an absorption pad)	The spray and absorbent pad applications reduced TNB* growth similarly after 6 h. During storage, control samples saw a 3-log increase, while the phage cocktail applications reduced TNB by 99% (2-log reduction).	[242]
mixed-leaf salad with carrot (20 °C/protective atmosphere)			Spray and absorbent pad applications showed no significant bacterial reduction at 6 h but significantly reduced TNB at 48 h. The absorbent pad was more effective on the food product. During storage, microbial counts in phage spray tests increased by 1 log, while both methods reduced TNB by 99.9% compared to controls.	
mixed-leaf salad with beetroot (20 °C/protective atmosphere)			The spray and absorbent pad had little effect on TNB at 6 h but significantly reduced growth at 48 h. Spraying was more effective on the product. Both methods reduced TNB by 99% (2-log reduction) compared to controls.	
washed spinach (20 °C/protective atmosphere)			The spray and absorbent pad significantly reduced bacterial growth at 6 h, but no inhibitory effect was observed at 24 h and 48 h.	
unwashed spinach (20 °C/protective atmosphere)			Applying the spray and absorbent pad to unwashed spinach significantly reduced TNB growth at 6 h and 24 h, but not at 48 h.	
broccoli sprouts (20 °C/protective atmosphere)	natural bacterial microbiota of the product	cocktail of 29 phages (spraying and an absorption pad)	The application of the spray and absorbent pad notably inhibited microbial growth in the product environment after 24 h. During storage, TNB in the control sample increased by less than half a logarithmic unit, whereas in the phage-treated samples, it decreased by a comparable amount.	[243]
spinach leaves (20 °C/protective atmosphere)			Both spraying and absorbent pad application effectively suppressed microbial growth in the product environment within 6 h compared to the control. The application of the phage cocktail on spinach leaves led to a reduction in TNB by half to nearly one log unit, depending on the method used.	
freshly squeezed carrot–celery juice (20 °C/protective atmosphere)		cocktail of 29 phages (phage suspension (5% of the volume of juice))	Throughout the 48 h storage period, TNB in the control sample remained largely unchanged. However, applying the phage suspension to the juice significantly reduced TNB within 6 h. During storage, TNB in the product treated with the phage mixture continued to decline steadily.	
fresh-cut mixed vegetables (iceberg lettuce, carrot, and purple cabbage) (20 °C/nitrogen gas)	*E. coli* strain K12	cocktail of coliphages (Escherichia phage SUT_E420, Escherichia phage SUT_E520, Escherichia phage SUT_E1520, and Escherichia phage SUT_E1620) (phage suspension)	When the coliphage cocktail was applied, no *E. coli* were detected in treated samples on day 0, showing a reduction of approximately 3.8 log CFU mL^−1^. However, *E. coli* levels gradually increased to 0.9–1.4 log CFU mL^−1^. Despite this, bacterial counts in treated samples remained significantly lower than in untreated samples throughout the experiment, with a 2.4 log CFU mL^−1^ reduction still observed by the end of day 3.	[244]
lettuce leaves (*Lactuca sativa var* iceberg) (no data)	*Enterobacter kobei* strain AG07E	bacteriophage FENT2 (single-phage treatment by immersion)	Immersing lettuce leaves in the phage suspension effectively reduced the presence of the *E. kobei* strain AG07E on their surface, resulting in a significant reduction of 1 ± 0.06 log.	[245]
**Bacterial Pathogens**				
**Food Matrices** **(Storage Conditions)**	**Bacteria**	**Phage/Phage Cocktail** **(Application Method)**	**Main Results**	**Reference**
radish sprouts (20 °C/normal atmosphere)	*S.* Virchow strain KKP 997	cocktail of four salmophages (KKP 3265, KKP 3266, KKP 3267, and KKP 3332) (spraying, and an absorption pad)	In all cases, the absorbent pad more effectively inhibited *Salmonella* growth in the food matrix, reducing it by up to 2 log units, compared to the spray method.	[246]
	*S.* Itami strain KKP 1001			
	*S.* Enteritidis strain KKP 3078			
	*S.* Typhimurium strain KKP 3079			
HHP-preserved carrot–mango–apple juice(4 °C)	*S. enterica* subsp. *enterica* serovar 6,8:l,-:1,7 strain KKP 1762	single or phage cocktail: Salmonella phage KKP 3822, Salmonella phage KKP 3829, Salmonella phage KKP 3830, and Salmonella phage KKP 3831 (phage suspension)	Applying either individual phages or a phage cocktail to juice significantly reduced *Salmonella* growth, except when Salmonella phage KKP 3830 was added to juice infected with *S. enterica* strain KKP 1762, where no significant reduction occurred. Pathogen growth was most effectively restricted by the phage targeting the specific bacterial strain. The phage cocktail reduced the growth of both bacterial pathogen strains by approximately one log unit (90%) by the end of refrigerated storage, compared to untreated control samples.	[247]
	*S.* Typhimurium strain KKP 3080			
raw carrot–apple juice (4 °C or 20 °C)	*S. enterica* subsp. *enterica* serovar 6,8:l,-:1,7 strain KKP 1762	phage cocktail: Salmonella phage KKP 3822, Salmonella phage KKP 3829, Salmonella phage KKP 3830, and Salmonella phage KKP 3831 (phage suspension)	Addition of a phage cocktail to juice infected with *S. enterica* strain KKP 1762 and storage at 4 °C for 24 h significantly reduced the pathogen level. For *S*. Typhimurium strain KKP 3080, a significant reduction was observed after 48 h. At 20 °C, control samples showed a one-fold increase in *Salmonella* counts after 24 h, while phage-treated samples showed a significant reduction. After two days at 20 °C or seven days at 4 °C, no *Salmonella* were detected in either control or phage-treated samples (strong pH changes during storage).	
	*S.* Typhimurium strain KKP 3080			
alfalfa sprout seeds (22 °C/in the dark)	*S.* Enteritidis strain S5-483, *S.* Newport strain S5-639, *S.* Muenchen strain S5-504, and *S.* Typhimurium strain S5-5336	phage cocktail (SE14, SE20, SF6) (single-phage treatment or repeated-phage treatment via washing)	The results indicate that *S*. Enteritidis was the most susceptible to both bacteriophage cocktails, showing a reduction of approximately 2.5 log CFU mL^−1^ on day 0 with cocktails SE14, SF5, and SF6. While *S. enterica* populations across all strains continued to grow despite daily bacteriophage applications, their growth rate was significantly lower compared to a single bacteriophage application. The extent of reduction varied depending on the *S. enterica* strain, but the findings suggest that repeated-phage treatment during sprout germination are more effective at reducing *S. enterica* populations than single-phage application.	[248]
		phage cocktail (SE14, SF5, SF6) (single-phage treatment or repeated-phage treatment via washing)		
green juice (4 °C and 25 °C)	Shiga toxin-producing *E. coli* O157:H7 (STEC) strain ATCC 43895	bacteriophage vB_ESM-pEJ01 (phage suspension)	The phage’s effectiveness was evaluated by measuring viable host cell counts and Shiga toxin genes (*stx1*, *stx2*) abundance. After 24 h at 4 °C, phage treatment reduced host cells by 1.5 log CFU mL^−1^. At 25 °C, a 2.7 log CFU mL^−1^ reduction occurred after 12 h, but bacterial regrowth was observed at 24 h, suggesting better efficacy at 4 °C. Phage-treated samples also showed a significant decrease in *stx* gene abundance at 4 °C after 24 h and at 25 °C after 12 h.	[249]
Romaine lettuce (10 °C)	*E. coli* O157 (STEC)	single coliphages (VE04, VE05, and VE07)	All tested phages successfully inhibited *E. coli* O157 on Romaine lettuce. After three days of storage at 10 °C, the log reduction compared to control groups ranged from 2.6 log CFU cm^−2^ to approximately 6 log CFU cm^−2^.	[250]
celery (4 °C)	*L. monocytogenes*	cocktail of three lytic phages: LMPC01, LMPC02, and LMPC03 (spraying)	During 7 days of refrigerated storage, the phage cocktail with MOI = 10 reduced *L. monocytogenes* by 2.2 log CFU g^−1^.	[251]
enoki mushroom (4 °C)			During 7 days of refrigerated storage, the phage cocktail with MOI = 10 reduced *L. monocytogenes* by 1.8 log CFU g^−1^.	
baby spinach (8 °C, 12 °C, and 25 °C)	*L. monocytogenes* strain 3053	phage vB_LmoH_P61 (spraying)	After 6 days of storage at 8, 12, and 25 °C, the concentration of *L. monocytogenes* decreased by 1.93, 2.06, and 3.3 log CFU g^−1^, respectively.	[252]
orange juice (4 °C)	*L. monocytogenes* strain LM008	phage vB-LmoM-SH3-3 (phage suspension)	Phage treatment resulted in an approximately 3.9-log decrease in *L. monocytogenes*.	[253]

## Data Availability

No new data were created or analyzed in this study. Data sharing is not applicable to this article.

## Abbreviations

The following abbreviations are used in this manuscript:

Abi	abortive infection system
ACE	accessory cholera enterotoxin
AIDS	acquired immunodeficiency syndrome
AMR	antimicrobial resistance
AMS	antibiotic stewardship
ARG	antibiotic resistance gene
BREX	bacteriophage exclusion
BVS	Bacterial Viruses Subcommittee
cAMP	cyclic adenosine monophosphate
Cas	CRISPR-associated genes
CDC	Centers for Disease Control and Prevention
CFU	colony-forming unit
CIP	clean-in-place
CRISPR	clustered, regularly interspaced short palindromic repeats
CT	cholera toxin
DHF	dihydrofolate
DISARM	defense island system associated with restriction modification
dsDNA	double-stranded deoxyribonucleic acid
dsRNA	double-stranded ribonucleic acid
ECDC	European Centre for Disease Prevention and Control
EFSA	European Food Safety Authority
EHEC	enterohemorrhagic Escherichia coli
EMA	European Medicines Agency
EU	European Union
FAO	Food and Agriculture Organization
FDA	Food and Drug Administration
FEEDAP	Panel on Additives and Products or Substances used in Animal Feed
GEI	genome island
GRAS	generally recognized as safe
HGT	horizontal gene transfer
HHP	high hydrostatic pressure
HIIET PAS	Ludwik Hirszfeld Institute of Immunology and Experimental Therapy of the Polish Academy of Sciences
HMC	glucosylated hydroxymethylcytosine
ICTV	International Committee on Taxonomy of Viruses
LAB	lactic acid bacteria
LPS	lipopolysaccharide
MDR	multidrug-resistant, multidrug resistance
MPF	minimally processed food
MRSA	methicillin-resistant Staphylococcus aureus
PABA	p–aminobenzoic acid
PAHO	Pan American Health Organization
PAI	pathogenicity island
PAM	protospacer-adjacent motif
PDR	pandrug resistance
PFU	plaque-forming unit
PIP	phage infection protein
qPCR	quantitative polymerase chain reaction
R-M	restriction–modification
ROS	reactive oxygen species
SCFA	short-chain fatty acid
Sie	superinfection exclusion mechanism
TA	toxin-antitoxin
TEM	transmission electron microscopy
THF	tetrahydrofolate
TNB	total number of bacteria
USDA	United States Department of Agriculture
WHO	World Health Organization
XDR	extensively drug resistance
ZOT	zonula occludens toxin

## References

[1] GBD 2016 Causes of Death Collaborators (2017). Global, regional, and national age–sex specific mortality for 264 causes of death, 1980–2016: A systematic analysis for the Global Burden of Disease Study 2016. Lancet.

[2] Vandevijvere S., De Ridder K., Fiolet T., Bel S., Tafforeau J. (2019). Consumption of ultra–processed food products and diet quality among children, adolescents and adults in Belgium. Eur. J. Nutr..

[3] Monteiro C.A., Cannon G., Levy R.B., Moubarac J.C., Louzada M.L., Rauber F., Khandpur N., Cediel G., Neri D., Martinez-Steele E. (2019). Ultra–processed foods: What they are and how to identify them. Public Health Nutr..

[4] Ng R., Sutradhar R., Yao Z., Wodchis W.P., Rosella L.C. (2020). Smoking, drinking, diet and physical activity—Modifiable lifestyle risk factors and their associations with age to first chronic disease. Int. J. Epidemiol..

[5] Jagannathan R., Patel S.A., Ali M.K., Narayan K.V. (2019). Global updates on cardiovascular disease mortality trends and attribution of traditional risk factors. Curr. Diabetes Rep..

[6] Juul F., Vaidean G., Parekh N. (2021). Ultra–processed foods and cardiovascular diseases: Potential mechanisms of action. Adv. Nutr..

[7] Monteiro C.A. (2009). Nutrition and health. The issue is not food, nor nutrients, so much as processing. Public Health Nutr..

[8] Petrus R.R., do Amaral Sobral P.J., Tadini C.C., Gonçalves C.B. (2021). The NOVA classification system: A critical perspective in food science. Trends Food Sci. Tech..

[9] Katidi A., Xanthopoulou S., Vlassopoulos A., Noutsos S., Priftis K., Kapsokefalou M. (2023). Food allergens in ultra–processed foods according to the NOVA classification system: A Greek branded food level analysis. Nutrients.

[10] Monteiro C.A., Cannon G., Moubarac J.C., Levy R.B., Louzada M.L.C., Jaime P.C. (2018). The UN decade of nutrition, the NOVA food classification and the trouble with ultra–processing. Public Health Nutr..

[11] Monteiro C.A., Cannon G., Levy R., Moubarac J.C., Jaime P., Martins A.P., Canella D., Louzada M., Parra D. (2016). NOVA. The star shines bright. World Nutr..

[12] De Corato U. (2020). Improving the shelf–life and quality of fresh and minimally–processed fruits and vegetables for a modern food industry: A comprehensive critical review from the traditional technologies into the most promising advancements. Crit. Rev. Food Sci..

[13] Meserole L., Iwu M.M., Wootton J.C. (2002). Health foods in anti–aging therapy: Reducers of physiological decline and degenerative diseases. Advances in Phytomedicine.

[14] Ragaert P., Verbeke W., Devlieghere F., Debevere J. (2004). Consumer perception and choice of minimally processed vegetables and packaged fruits. Food Qual. Prefer..

[15] Knorr D., Watzke H. (2019). Food processing at a crossroad. Front. Nutr..

[16] Monteiro C.A., Cannon G., Lawrence M., da Costa Louzada M.L., Machado P.P. (2019). Ultra–Processed Foods, Diet Quality, and Health Using the Nova Classification System.

[17] Sarker A., Grift T.E. (2021). Bioactive properties and potential applications of Aloe vera gel edible coating on fresh and minimally processed fruits and vegetables: A review. J. Food Meas. Charact..

[18] Monteiro C.A., Levy R.B., Claro R.M., Castro I.R.R.D., Cannon G. (2010). A new classification of foods based on the extent and purpose of their processing. Cad. Saude Publica.

[19] Bansal V., Siddiqui M.W., Rahman M.S., Siddiqui M., Rahman M. (2015). Minimally processed foods: Overview. Minimally Processed Foods: Technologies for Safety, Quality, and Convenience.

[20] Agriopoulou S., Stamatelopoulou E., Sachadyn-Król M., Varzakas T. (2020). Lactic acid bacteria as antibacterial agents to extend the shelf life of fresh and minimally processed fruits and vegetables: Quality and safety aspects. Microorganisms.

[21] Siddiqui M.W., Chakraborty I., Ayala-Zavala J.F., Dhua R.S. (2011). Advances in minimal processing of fruits and vegetables: A review. J. Sci. Ind. Res..

[22] Mostafidi M., Sanjabi M.R., Shirkhan F., Zahedi M.T. (2020). A review of recent trends in the development of the microbial safety of fruits and vegetables. Trends Food Sci. Tech..

[23] Liu X., Le Bourvellec C., Yu J., Zhao L., Wang K., Tao Y., Renard C.M.G.C., Hu Z. (2022). Trends and challenges on fruit and vegetable processing: Insights into sustainable, traceable, precise, healthy, intelligent, personalized and local innovative food products. Trends Food Sci. Tech..

[24] Al-Khusaibi M., Al-Habsi N., Shafiur Rahman M., Al-Khusaibi M., Al-Habsi N., Shafiur Rahman M. (2019). Traditional Foods.

[25] Endo H., Miyazaki K., Ose K., Imahori Y. (2019). Hot water treatment to alleviate chilling injury and enhance ascorbate-glutathione cycle in sweet pepper fruit during postharvest cold storage. Sci. Hortic..

[26] Terefe N.S., Buckow R., Versteeg C. (2014). Quality–related enzymes in fruit and vegetable products: Effects of novel food processing technologies, part 1: High–pressure processing. Crit. Rev. Food Sci. Nutr..

[27] Tapia M.S., Alzamora S.M., Chirife J., Barbosa-Cánovas G.V., Fontana A.J., Schmidt S.J., Labuza T.P. (2020). Effects of water activity (a_w_) on microbial stability as a hurdle in food preservation. Water Activity in Foods: Fundamentals and Applications.

[28] George A.S., Simko I., Brandl M.T. (2025). *Escherichia coli* O157:H7 multiplication in the latex of diverse lettuce genotypes is negatively correlated with plant peroxidase activity. Int. J. Food Microbiol..

[29] Liu H., Li Z., Zhang X., Liu Y., Hu J., Yang C., Zhao X. (2021). The effects of ultrasound on the growth, nutritional quality and microbiological quality of sprouts. Trends Food Sci. Technol..

[30] Shymialevich D., Wójcicki M., Wardaszka A., Świder O., Sokołowska B., Błażejak S. (2023). Application of lytic bacteriophages and their enzymes to reduce saprophytic bacteria isolated from minimally processed plant–based food products—In vitro studies. Viruses.

[31] Ramos B., Brandão T.R.S., Teixeira P., Silva C.L.M. (2020). Biopreservation approaches to reduce *Listeria monocytogenes* in fresh vegetables. Food Microbiol..

[32] Yildiz H., Karatas N. (2018). Microbial exopolysaccharides: Resources and bioactive properties. Process Biochem..

[33] George F., Daniel C., Thomas M., Singer E., Guilbaud A., Tessier F.J., Revol-Junelles A.M., Borges F., Foligné B. (2018). Occurrence and dynamism of lactic acid bacteria in distinct ecological niches: A multifaceted functional health perspective. Front. Microbiol..

[34] Siroli L., Patrignani F., Serrazanetti D.I., Gardini F., Lanciotti R. (2015). Innovative strategies based on the use of bio–control agents to improve the safety, shelf–life and quality of minimally processed fruits and vegetables. Trends Food Sci. Tech..

[35] Sharma V., Ranveer R.C., Jain N., Aseri G.K. (2019). Bacteriocins: Production, different strategies of purification and applications. Int. J. Res. Pharm. Sci..

[36] Barbosa A.A., Mantovani H.C., Jain S. (2017). Bacteriocins from lactic acid bacteria and their potential in the preservation of fruit products. Crit. Rev. Biotechnol..

[37] Browne K., Chakraborty S., Chen R., Willcox M.D., Black D.S., Walsh W.R., Kumar N. (2020). A new era of antibiotics: The clinical potential of antimicrobial peptides. Int. J. Mol. Sci..

[38] Ellinger E., Chauvier A., Romero R.A., Liu Y., Ray S., Walter N.G. (2023). Riboswitches as therapeutic targets: Promise of a new era of antibiotics. Expert Opin. Ther. Tar..

[39] Low C.X., Tan L.T.-H., Ab Mutalib N.-S., Pusparajah P., Goh B.-H., Chan K.-G., Letchumanan V., Lee L.-H. (2021). Unveiling the impact of antibiotics and alternative methods for animal husbandry: A review. Antibiotics.

[40] Sakkas H., Bozidis P., Ilia A., Mpekoulis G., Papadopoulou C. (2019). Antimicrobial resistance in bacterial pathogens and detection of carbapenemases in Klebsiella pneumoniae isolates from hospital wastewater. Antibiotics.

[41] Chandra P., Unnikrishnan M.K., Vandana K.E., Mukhopadhyay C., Dinesh Acharya U., Surulivel Rajan M., Rajesh V. (2021). Antimicrobial resistance and the post antibiotic era: Better late than never effort. Expert. Opin. Drug Saf..

[42] Priyadharsini R.P. (2019). Antibiotic resistance: What is there in past, present and future?. J. Young Pharm..

[43] Kose A., Colak C. (2021). Knowledge and awareness of physicians about rational antibiotic use and antimicrobial resistance before and after graduation: A cross–sectional study conducted in Malatya Province in Turkey. Infect. Drug Resist..

[44] Godziszewska J., Guzek D., Głąbski K., Wierzbicka A. (2016). Mobilna antybiotykooporność—O rozprzestrzenianiu się genów determinujących oporność bakterii poprzez produkty spożywcze [Mobile antibiotic resistance—The spread of genes determining the resistance of bacteria through food products]. Postep. Hig. Med. Dosw..

[45] Ben Y., Fu C., Hu M., Liu L., Wong M.H., Zheng C. (2019). Human health risk assessment of antibiotic resistance associated with antibiotic residues in the environment: A review. Environ. Res..

[46] Vikesland P.J., Pruden A., Alvarez P.J.J., Aga D., Bürgmann H., Li X.D., Manaia C.M., Nambi I., Wigginton K., Zhang T. (2017). Toward a comprehensive strategy to mitigate dissemination of environmental sources of antibiotic resistance. Environ. Sci. Technol..

[47] Vinayamohan P.G., Pellissery A.J., Venkitanarayanan K. (2022). Role of horizontal gene transfer in the dissemination of antimicrobial resistance in food animal production. Curr. Opin. Food Sci..

[48] Mole B. (2013). MRSA: Farming up trouble. Nature.

[49] Wang N., Yang X., Jiao S., Zhang J., Ye B., Gao S. (2014). Sulfonamide–resistant bacteria and their resistance genes in soils fertilized with manures from Jiangsu Province, Southeastern China. PLoS ONE.

[50] Llor C., Bjerrum L. (2014). Antimicrobial resistance: Risk associated with antibiotic overuse and initiatives to reduce the problem. Ther. Adv. Drug Saf..

[51] Van T.T.H., Yidana Z., Smooker P.M., Coloe P.J. (2020). Antibiotic use in food animals worldwide, with a focus on Africa: Pluses and minuses. J. Glob. Antimicrob. Resist..

[52] Bisht R., Katiyar A., Singh R., Mittal P. (2009). Antibiotic resistance—A global issue of concern. Asian J. Pharm. Clin. Res..

[53] Ma F., Xu S., Tang Z., Li Z., Zhang L. (2021). Use of antimicrobials in food animals and impact of transmission of antimicrobial resistance on humans. Biosaf. Health.

[54] Chaudhary A.S. (2016). A review of global initiatives to fight antibiotic resistance and recent antibiotics’ discovery. Acta Pharm. Sin. B.

[55] Habib F., Roy D., Nity M. (2021). Effect of promoter region sequence variations in relation to antibiotic resistance of methicillin–resistant *Staphylococcus aureus*. J. Med. Clin. Res. Rev..

[56] Bhat R.A.H., Altinok I. (2023). Antimicrobial resistance (AMR) and alternative strategies for combating AMR in aquaculture. Turk. J. Fish. Aquat. Sci..

[57] Oluwafemi R.A., Olawale I., Alagbe J.O. (2020). Recent trends in the utilization of medicinal plants as growth promoters in poultry nutrition—A review. Res. Agric. Vet. Sci..

[58] Cuong N.V., Kiet B.T., Hien V.B., Truong B.D., Phu D.H., Thwaites G., Choisy M., Carrique-Mas J. (2021). Antimicrobial use through consumption of medicated feeds in chicken flocks in the Mekong Delta of Vietnam: A three–year study before a ban on antimicrobial growth promoters. PLoS ONE.

[59] Council of the European Union Programme of Community Action in the Field of Public Health (2003 to 2008). https://eur-lex.europa.eu/legal-content/EN/ALL/?uri=LEGISSUM:c11503b.

[60] Narodowy Program Ochrony Antybiotyków [National Antibiotic Protection Program]. https://antybiotyki.edu.pl/.

[61] Taati Moghadam M., Khoshbayan A., Chegini Z., Farahani I., Shariati A. (2020). Bacteriophages, a new therapeutic solution for inhibiting multidrug–resistant bacteria causing wound infection: Lesson from animal models and clinical trials. Drug Des. Dev. Ther..

[62] Broncano-Lavado A., Santamaría-Corral G., Esteban J., García-Quintanilla M. (2021). Advances in bacteriophage therapy against relevant multidrug–resistant pathogens. Antibiotics.

[63] Nikolich M.P., Filippov A.A. (2020). bacteriophage therapy: Developments and directions. Antibiotics.

[64] Hyldgaard M. (2020). Mechanisms of Action, Resistance, and Stress Adaptation. Antimicrobials in Food.

[65] Etebu E., Arikekpar I. (2016). Antibiotics: Classification and mechanisms of action with emphasis on molecular perspectives. Int. J. Appl. Microbiol. Biotechnol. Res..

[66] Bitew Z., Amare M. (2020). Recent reports on electrochemical determination of selected antibiotics in pharmaceutical formulations: A mini review. Electrochem. Commun..

[67] Al-Hasani H.M., Al-Rubaye D.S., Abdelhameed A. (2023). The emergence of multidrug–resistant (MDR), extensively drug–resistant (XDR), and pandrug–resistant (PDR) in Iraqi clinical isolates of *Escherichia coli*. J. Popul. Ther. Clin. Pharmacol..

[68] Rafailidis P.I., Kofteridis D. (2022). Proposed amendments regarding the definitions of multidrug–resistant and extensively drug–resistant bacteria. Expert Rev. Anti Infect. Ther..

[69] Alkofide H., Alhammad A.M., Alruwaili A., Aldemerdash A., Almangour T.A., Alsuwayegh A., Almoqbel D., Albati A., Alsaud A., Enani M. (2020). Multidrug–resistant and extensively drug–resistant *Enterobacteriaceae*: Prevalence, treatments, and outcomes—A retrospective cohort study. Infect. Drug Resist..

[70] Hasan T.H., Al-Harmoosh R.A. (2020). Mechanisms of antibiotics resistance in bacteria. Syst. Rev. Pharm..

[71] Kakoullis L., Papachristodoulou E., Chra P., Panos G. (2021). Mechanisms of antibiotic resistance in important Gram–positive and Gram–negative pathogens and novel antibiotic solutions. Antibiotics.

[72] Odonkor S.T., Addo K.K. (2011). Bacteria resistance to antibiotics: Recent trends and challenges. Int. J. Biol. Med. Res..

[73] Mancuso G., Midiri A., Gerace E., Biondo C. (2021). Bacterial antibiotic resistance: The most critical pathogens. Pathogens.

[74] Naskar A., Kim K.S. (2019). Nanomaterials as delivery vehicles and components of new strategies to combat bacterial infections: Advantages and limitations. Microorganisms.

[75] Thandar M., Lood R., Winer B.Y., Deutsch D.R., Euler C.W., Fischetti V.A. (2016). Novel engineered peptides of a phage lysin as effective antimicrobials against multidrug–resistant *Acinetobacter baumannii*. Antimicrob. Agents Chemother..

[76] Lin D.M., Koskella B., Lin H.C. (2017). Phage therapy: An alternative to antibiotics in the age of multi–drug resistance. World J. Gastrointest. Pharmacol. Ther..

[77] Makumi A., Mhone A.L., Odaba J., Guantai L., Svitek N. (2021). Phages for Africa: The potential benefit and challenges of phage therapy for the livestock sector in Sub–Saharan Africa. Antibiotics.

[78] Pires D.P., Costa A.R., Pinto G., Meneses L., Azeredo J. (2020). Current challenges and future opportunities of phage therapy. FEMS Microbiol. Rev..

[79] Żaczek M., Weber-Dąbrowska B., Międzybrodzki R., Łusiak-Szelachowska M., Górski A. (2020). Phage therapy in Poland—A centennial journey to the first ethically approved treatment facility in Europe. Front. Microbiol..

[80] Dąbrowska K. (2019). Phage therapy: What factors shape phage pharmacokinetics and bioavailability? Systematic and critical review. Med. Res. Rev..

[81] Rostkowska O.M., Międzybrodzki R., Miszewska-Szyszkowska D., Górski A., Durlik M. (2021). Treatment of recurrent urinary tract infections in a 60–year–old kidney transplant recipient. The use of phage therapy. Transpl. Infect. Dis..

[82] Mahony J., Casey E., van Sinderen D. (2020). The impact and applications of phages in the food industry and agriculture. Viruses.

[83] Gientka I., Wójcicki M., Żuwalski A.W., Błażejak S. (2021). Use of phage cocktail for improving the overall microbiological quality of sprouts—Two methods of application. Appl. Microbiol..

[84] Xu Y. (2021). Phage and phage lysins: New era of bio–preservatives and food safety agents. J. Food Sci..

[85] Wójcik E.A., Stańczyk M., Wojtasik A., Kowalska J.D., Nowakowska M., Łukasiak M., Bartnicka M., Kazimierczak J., Dastych J. (2020). Comprehensive evaluation of the safety and efficacy of BAFASAL^®^ bacteriophage preparation for the reduction of *Salmonella* in the food chain. Viruses.

[86] Kuek M., McLean S.K., Palombo E.A. (2022). Application of bacteriophages in food production and their potential as biocontrol agents in the organic farming industry. Biol. Control.

[87] Wójcicki M., Błażejak S., Gientka I., Brzezicka K. (2019). The concept of using bacteriophages to improve the microbiological quality of minimally processed foods. Acta Sci. Pol. Technol. Aliment..

[88] Naureen Z., Dautaj A., Anpilogov K., Camilleri G., Dhuli K., Tanzi B., Maltese P.E., Cristofoli F., De Antoni L., Beccari T. (2020). Bacteriophages presence in nature and their role in the natural selection of bacterial populations. Acta Biomed..

[89] Holtappels D., Alfenas-Zerbini P., Koskella B. (2023). Drivers and consequences of bacteriophage host range. FEMS Microbiol. Rev..

[90] Ali A., Jørgensen J.S., Lamont R.F. (2022). The contribution of bacteriophages to the aetiology and treatment of the bacterial vaginosis syndrome. Fac. Rev..

[91] Nazarov P.A., Baleev D.N., Ivanova M.I., Sokolova L.M., Karakozova M.V. (2020). Infectious plant diseases: Etiology, current status, problems and prospects in plant protection. Acta Naturae.

[92] Kurilovich E., Geva-Zatorsky N. (2025). Effects of bacteriophages on gut microbiome functionality. Gut Microbes.

[93] Brussow H., Canchaya C., Hardt W.D. (2004). Phages and the evolution of bacterial pathogens: From genomic rearrangements to lysogenic conversion. Microbiol. Mol. Biol. Rev..

[94] Dion M.B., Oechslin F., Moineau S. (2020). Phage diversity, genomics and phylogeny. Nat. Rev. Microbiol..

[95] Ramos-Vivas J., Superio J., Galindo-Villegas J., Acosta F. (2021). Phage therapy as a focused management strategy in aquaculture. Int. J. Mol. Sci..

[96] Siddell S.G., Smith D.B., Adriaenssens E., Alfenas-Zerbini P., Dutilh B.E., Garcia M.L., Junglen S., Krupovic M., Kuhn J.H., Lambert A.J. (2023). Virus taxonomy and the role of the International Committee on Taxonomy of Viruses (ICTV). J. Gen. Virol..

[97] Van Regenmortel M.H., Ackermann H.W., Calisher C.H., Dietzgen R.G., Horzinek M.C., Keil G.M., Mahy B.W.J., Martelli G.P., Murphy F.A., Pringle C. (2013). Virus species polemics: 14 senior virologists oppose a proposed change to the ICTV definition of virus species. Arch. Virol..

[98] Adriaenssens E., Brister J.R. (2017). How to name and classify your phage: An informal guide. Viruses.

[99] Lefkowitz E.J., Dempsey D.M., Hendrickson R.C., Orton R.J., Siddell S.G., Smith D.B. (2017). Virus taxonomy: The database of the International Committee on Taxonomy of Viruses (ICTV). Nucleic Acids Res..

[100] Siddell S.G., Walker P.J., Lefkowitz E.J., Mushegian A.R., Dutilh B.E., Harrach B., Harrison R.L., Junglen S., Knowles N.J., Kropinski A.M. (2020). Binomial nomenclature for virus species: A consultation. Arch. Virol..

[101] Zerbini F.M., Siddell S.G., Mushegian A.R., Walker P.J., Lefkowitz E.J., Adriaenssens E.M., Alfenas-Zerbini P., Dutilh B.E., García M.L., Junglen S. (2022). Differentiating between viruses and virus species by writing their names correctly. Arch. Virol..

[102] Turner D., Shkoporov A.N., Lood C., Millard A.D., Dutilh B.E., Alfenas-Zerbini P., van Zyl L.J., Aziz R.K., Oksanen H.M., Poranen M.M. (2023). Abolishment of morphology–based taxa and change to binomial species names: 2022 taxonomy update of the ICTV bacterial viruses subcommittee. Arch. Virol..

[103] Nami Y., Imeni N., Panahi B. (2021). Application of machine learning in bacteriophage research. BMC Microbiol..

[104] Yang Y., Fan C., Zhao Q. (2020). Recent advances on the machine learning methods in identifying phage virion proteins. Curr. Bioinform..

[105] Lood C., Boeckaerts D., Stock M., De Baets B., Lavigne R., van Noort V., Briers Y. (2022). Digital phagograms: Predicting phage infectivity through a multilayer machine learning approach. Curr. Opin. Virol..

[106] Boeckaerts D., Stock M., De Baets B., Briers Y. (2022). Identification of phage receptor–binding protein sequences with hidden Markov models and an extreme gradient boosting classifier. Viruses.

[107] Andrade-Martínez J.S., Camelo Valera L.C., Chica Cardenas L.A., Forero-Junco L., López-Leal G., Moreno-Gallego J.L., Rangel-Pineros G., Reyes A. (2022). Computational tools for the analysis of uncultivated phage genomes. Microbiol. Mol. Biol. Rev..

[108] Jiang J.Z., Yuan W.G., Shang J., Shi Y.H., Yang L.L., Liu M., Zhu P., Jin T., Sun Y., Yuan L.H. (2023). Virus classification for viral genomic fragments using PhaGCN2. Brief. Bioinform..

[109] Cebeci A., Türe M., Alemdağ M., Altinok I. (2023). Whole genome sequence of a novel bacteriophage APT65 infecting *Aeromonas hydrophila*. PHAGE.

[110] Tynecki P., Guziński A., Kazimierczak J., Jadczuk M., Dastych J., Onisko A. (2020). PhageAI—Bacteriophage life cycle recognition with machine learning and natural language processing. bioRXiv.

[111] Verheust C., Pauwels K., Mahillon J., Helinski D.R., Herman P. (2010). Contained use of bacteriophages: Risk assessment and biosafety recommendations. Appl. Biosaf..

[112] Sanz-Gaitero M., Seoane-Blanco M., van Raaij M.J., Harper D.R., Abedon S.T., Burrowes B.H., McConville M.L. (2021). Structure and function of bacteriophages. Bacteriophages.

[113] Aksyuk A.A., Rossmann M.G. (2011). Bacteriophage assembly. Viruses.

[114] Chaitanya K.V. (2019). Structure and organization of virus genomes. Genome and Genomics: From Archaea to Eukaryotes.

[115] Jończyk E., Kłak M., Międzybrodzki R., Górski A. (2011). The influence of external factors on bacteriophages. Folia Microbiol..

[116] Orlova E., Kurtboke I. (2012). Bacteriophages and their structural organisation. Bacteriophages.

[117] Lauman P., Dennis J.J. (2021). Advances in phage therapy: Targeting the *Burkholderia cepacia* complex. Viruses.

[118] Schmalstig A.A., Freidy S., Hanafin P.O., Braunstein M., Rao G.G. (2021). Reapproaching old treatments: Considerations for PK/PD studies on phage therapy for bacterial respiratory infections. Clin. Pharmacol. Ther..

[119] Liu Y., Demina T.A., Roux S., Aiewsakun P., Kazlauskas D., Simmonds P., Prangishvili D., Oksanen H.M., Krupovic M. (2021). Diversity, taxonomy, and evolution of archaeal viruses of the class Caudoviricetes. PLoS Biol..

[120] Dutilh B.E., Varsani A., Tong Y., Simmonds P., Sabanadzovic S., Rubino L., Roux S., Muñoz A.R., Lood C., Lefkowitz E.J. (2021). Perspective on taxonomic classification of uncultivated viruses. Curr. Opin. Virol..

[121] International Committee on Taxonomy of Viruses. https://ictv.global/.

[122] Sieiro C., Areal-Hermida L., Pichardo-Gallardo Á., Almuiña-González R., de Miguel T., Sánchez S., Sánchez-Pérez Á., Villa T.G. (2020). A hundred years of bacteriophages: Can phages replace antibiotics in agriculture and aquaculture?. Antibiotics.

[123] Zhang M., Zhang T., Yu M., Chen Y.-L., Jin M. (2022). The life cycle transitions of temperate phages: Regulating factors and potential ecological implications. Viruses.

[124] Leprince A., Mahillon J. (2023). Phage adsorption to Gram–positive bacteria. Viruses.

[125] Dupont K., Janzen T., Vogensen F.K., Josephsen J., Stuer-Lauridsen B. (2004). Identification of *Lactococcus lactis* genes required for bacteriophage adsorption. Appl. Environ. Microb..

[126] Yamaki S., Yamazaki K., Kawai Y. (2022). Broad host range bacteriophage, EscoHU1, infecting *Escherichia coli* O157:H7 and *Salmonella enterica*: Characterization, comparative genomics, and applications in food safety. Int. J. Food Microbiol..

[127] Golonka R., Yeoh B.S., Vijay-Kumar M. (2019). The iron tug–of–war between bacterial siderophores and innate immunity. J. Innate Immun..

[128] Dunne M., Hupfeld M., Klumpp J., Loessner M.J. (2018). Molecular basis of bacterial host interactions by Gram–positive targeting bacteriophages. Viruses.

[129] Stone E., Campbell K., Grant I., McAuliffe O. (2019). Understanding and exploiting phage–host interactions. Viruses.

[130] Millen A.M., Romero D.A. (2016). Genetic determinants of lactococcal c2 viruses for host infection and their role in phage evolution. J. Gen. Virol..

[131] Chevallereau A., Pons B.J., van Houte S., Westra E.R. (2022). Interactions between bacterial and phage communities in natural environments. Nat. Rev. Microbiol..

[132] Correa A.M.S., Howard-Varona C., Coy S.R., Buchan A., Sullivan M.B., Weitz J.S. (2021). Revisiting the rules of life for viruses of microorganisms. Nat. Rev. Microbiol..

[133] Mäntynen S., Laanto E., Oksanen H.M., Poranen M.M., Díaz-Muñoz S.L. (2021). Black box of phage–bacterium interactions: Exploring alternative phage infection strategies. Open Biol..

[134] Luong T., Salabarria A.C., Roach D.R. (2020). Phage therapy in the resistance era: Where do we stand and where are we going?. Clin. Ther..

[135] Endersen L., Coffey A. (2020). The use of bacteriophages for food safety. Curr. Opin. Food Sci..

[136] Popescu M., Van Belleghem J.D., Khosravi A., Bollyky P.L. (2021). Bacteriophages and the immune system. Ann. Rev. Virol..

[137] Samtlebe M., Denis S., Chalancon S., Atamer Z., Wagner N., Neve H., Franz C., Schmidt H., Blanquet-Diot S., Hinrichs J. (2018). Bacteriophages as modulator for the human gut microbiota: Release from dairy food systems and survival in a dynamic human gastrointestinal model. LWT Food Sci. Technol..

[138] Blanco-Picazo P., Fernández-Orth D., Brown-Jaque M., Miró E., Espinal P., Rodríguez-Rubio L., Muniesa M., Navarro F. (2020). Unravelling the consequences of the bacteriophages in human samples. Sci. Rep..

[139] Podlacha M., Grabowski Ł., Kosznik-Kwaśnicka K., Zdrojewska K., Stasiłojć M., Węgrzyn G., Węgrzyn A. (2021). Interactions of bacteriophages with animal and human organisms—Safety issues in the light of phage therapy. Int. J. Mol. Sci..

[140] Średnicka P., Roszko M.Ł., Popowski D., Kowalczyk M., Wójcicki M., Emanowicz P., Szczepańska M., Kotyrba D., Juszczuk-Kubiak E. (2023). Effect of in vitro cultivation on human gut microbiota composition using 16S rDNA amplicon sequencing and metabolomics approach. Sci. Rep..

[141] Pham V.T., Dold S., Rehman A., Bird J.K., Steinert R.E. (2021). Vitamins, the gut microbiome and gastrointestinal health in humans. Nutr. Res..

[142] Lippert K., Kedenko L., Antonielli L., Kedenko I., Gemeier C., Leitner M., Kautzky-Willer A., Paulweber B., Hackl E. (2017). Gut microbiota dysbiosis associated with glucose metabolism disorders and the metabolic syndrome in older adults. Benef. Microbes..

[143] Zhu S., Jiang Y., Xu K., Cui M., Ye W., Zhao G., Jin L., Chen X. (2020). The progress of gut microbiome research related to brain disorders. J. Neuroinflamm..

[144] Rowan-Nash A.D., Korry B.J., Mylonakis E., Belenky P. (2019). Cross–domain and viral interactions in the microbiome. Microbiol. Mol. Biol. Rev..

[145] Hsu C.L., Duan Y., Fouts D.E., Schnabl B. (2021). Intestinal virome and therapeutic potential of bacteriophages in liver disease. J. Hepatol..

[146] Manrique P., Dills M., Young M.J. (2017). The human gut phage community and its implications for health and disease. Viruses.

[147] Rybicka I., Kaźmierczak Z. (2025). The human phageome: Niche-specific distribution of bacteriophages and their clinical implications. Appl. Environ. Microbiol..

[148] Townsend E.M., Kelly L., Muscatt G., Box J.D., Hargraves N., Lilley D., Jameson E. (2021). The human gut phageome: Origins and roles in the human gut microbiome. Front. Cell. Infect. Microbiol..

[149] Tariq M.A., Everest F.L.C., Cowley L.A., De Soyza A., Holt G.S., Bridge S.H., Perry A., Perry J.D., Bourke S.J., Cummings S.P. (2015). A metagenomic approach to characterize temperate bacteriophage populations from Cystic Fibrosis and non–Cystic Fibrosis bronchiectasis patients. Front. Microbiol..

[150] Aleshkin A.V., Ershova O.N., Volozhantsev N.V., Svetoch E.A., Popova A.V., Rubalskii E.O., Borzilov A.I., Aleshkin V.A., Afanas’ev S.S., Karaulov A.V. (2016). Phagebiotics in treatment and prophylaxis of healthcare–associated infections. Bacteriophage.

[151] Jończyk-Matysiak E., Weber-Dąbrowska B., Owczarek B., Międzybrodzki R., Łusiak-Szelachowska M., Łodej N., Górski A. (2017). Phage–phagocyte interactions and their implications for phage application as therapeutics. Viruses.

[152] Federici S., Nobs S.P., Elinav E. (2021). Phages and their potential to modulate the microbiome and immunity. Cell. Mol. Immunol..

[153] Zimecki M., Weber-Dąbrowska B., Łusiak-Szelachowska M., Mulczyk M., Boratyński J., Poźniak G., Syper D., Górski A. (2003). Bacteriophages provide regulatory signals in mitogen–induced murine splenocyte proliferation. Cell. Mol. Biol. Lett..

[154] Shuwen H., Kefeng D. (2022). Intestinal phages interact with bacteria and are involved in human diseases. Gut Microbes.

[155] Cieślik M., Bagińska N., Jończyk-Matysiak E., Węgrzyn A., Węgrzyn G., Górski A. (2021). Temperate bacteriophages—The powerful indirect modulators of eukaryotic cells and immune functions. Viruses.

[156] Jaglan A.B., Anand T., Verma R., Vashisth M., Virmani N., Bera B.C., Vaid R.K., Tripathi B.N. (2022). Tracking the phage trends: A comprehensive review of applications in therapy and food production. Front. Microbiol..

[157] Rai S., Kaur B., Singh P., Singh A., Benjakul S., Vijay Kumar Reddy S., Nagar V., Tyagi A. (2023). Perspectives on phage therapy for health management in aquaculture. Aquacult. Int..

[158] Pradal I., Casado A., del Rio B., Rodriguez-Lucas C., Fernandez M., Alvarez M.A., Ladero V. (2023). Enterococcus faecium bacteriophage vB_EfaH_163, a new member of the *Herelleviridae* family, reduces the mortality associated with an *E. faecium vanR* clinical isolate in a *Galleria mellonella* animal model. Viruses.

[159] Arumugam S.N., Manohar P., Sukumaran S., Sadagopan S., Loh B., Leptihn S., Nachimuthu R. (2022). Antibacterial efficacy of lytic phages against multidrug–resistant *Pseudomonas aeruginosa* infections in bacteraemia mice models. BMC Microbiol..

[160] Amankwah S., Abdella K., Kassa T. (2021). Bacterial biofilm destruction: A focused review on the recent use of phage–based strategies with other antibiofilm agents. Nanotechnol. Sci. Appl..

[161] Liu S., Lu H., Zhang S., Shi Y., Chen Q. (2022). Phages against pathogenic bacterial biofilms and biofilm–based infections: A review. Pharmaceutics.

[162] Blasco L., Bleriot I., González de Aledo M., Fernández-García L., Pacios O., Oliveira H., López M., Ortiz-Cartagena C., Fernández-Cuenca F., Pascual Á. (2022). Development of an anti—*Acinetobacter baumannii* biofilm phage cocktail: Genomic adaptation to the host. Antimicrob. Agents Chemother..

[163] Verbeken G., Pirnay J.P. (2021). European regulatory aspects of phage therapy: Magistral phage preparations. Curr. Opin. Virol..

[164] Zhou Y., Li L., Han K., Wang L., Cao Y., Ma D., Wang X. (2022). A polyvalent broad–spectrum Escherichia phage *Tequatrovirus* EP01 capable of controlling *Salmonella* and *Escherichia coli* contamination in foods. Viruses.

[165] Ortiz Charneco G., de Waal P.P., van Rijswijck I.M., van Peij N.N., van Sinderen D., Mahony J. (2023). Bacteriophages in the dairy industry: A problem solved?. Annu. Rev. Food Sci. Technol..

[166] Olejnik-Schmidt A., Pietrzak B., Kawacka I., Malak K., Wawrzyniak W., Schmidt M. (2021). A simple method for assessing diversity and dynamics of microbial community: Comparison of dairy phages from industrial and spontaneous fermentation. Appl. Sci..

[167] Guo Y., Li J., Islam M.S., Yan T., Zhou Y., Liang L., Connerton I.F., Deng K., Li J. (2021). Application of a novel phage vB_SalS–LPSTLL for the biological control of *Salmonella* in foods. Food Res. Int..

[168] Cristobal-Cueto P., García-Quintanilla A., Esteban J., García-Quintanilla M. (2021). Phages in food industry biocontrol and bioremediation. Antibiotics.

[169] Chen Q., Dharmaraj T., Cai P.C., Burgener E.B., Haddock N.L., Spakowitz A.J., Bollyky P.L. (2022). Bacteriophage and bacterial susceptibility, resistance, and tolerance to antibiotics. Pharmaceutics.

[170] Pfeifer E., Bonnin A.R., Rocha E.P. (2022). Phage–plasmids spread antibiotic resistance genes through infection and lysogenic conversion. mBio.

[171] Michaelis C., Grohmann E. (2023). Horizontal gene transfer of antibiotic resistance genes in biofilms. Antibiotics.

[172] Gummalla V.S., Zhang Y., Liao Y.-T., Wu V.C.H. (2023). The role of temperate phages in bacterial pathogenicity. Microorganisms.

[173] Wang Y., Deng J., Ren J., Liang L., Li J., Niu S., Wu X., Zhao Y., Gao S., Yan F. (2022). RAP44 phage integrase–guided 50K genomic island integration in *Riemerella anatipestifer*. Front. Vet. Sci..

[174] Mai-Prochnow A., Hui J.G., Kjelleberg S., Rakonjac J., McDougald D., Rice S.A. (2015). Big things in small packages: The genetics of filamentous phage and effects on fitness of their host. FEMS Microbiol. Rev..

[175] Khouadja S., Roque A., Gonzalez M., Furones D. (2022). *Vibrio* pathogenicity island and phage CTX genes in *Vibrio alginolyticus* isolated from different aquatic environments. J. Water Health.

[176] Chen D., Liang Z., Ren S., Alali W., Chen L. (2022). Rapid and visualized detection of virulence–related genes of *Vibrio cholerae* in water and aquatic products by loop–mediated isothermal amplification. J. Food Prot..

[177] Faruque S.M., Mekalanos J.J. (2012). Phage–bacterial interactions in the evolution of toxigenic *Vibrio cholerae*. Virulence.

[178] Hibstu Z., Belew H., Akelew Y., Mengist H.M. (2022). Phage therapy: A different approach to fight bacterial infections. Biol. Targets Ther..

[179] Azam A.H., Tanji Y. (2019). Bacteriophage–host arm race: An update on the mechanism of phage resistance in bacteria and revenge of the phage with the perspective for phage therapy. Appl. Microbiol. Biotechnol..

[180] Jariah R.O.A., Hakim M.S. (2019). Interaction of phages, bacteria, and the human immune system: Evolutionary changes in phage therapy. Rev. Med. Virol..

[181] Isaev A.B., Musharova O.S., Severinov K.V. (2021). Microbial arsenal of antiviral defenses—Part I. Biochemistry.

[182] Abdelsattar A.S., Dawooud A., Rezk N., Makky S., Safwat A., Richards P.J., El-Shibiny A. (2021). How to train your phage: The recent efforts in phage training. Biologics.

[183] Kelly A., Arrowsmith T.J., Went S.C., Blower T.R. (2023). Toxin–antitoxin systems as mediators of phage defence and the implications for abortive infection. Cur. Opin. Microbiol..

[184] Lopatina A., Tal N., Sorek R. (2020). Abortive infection: Bacterial suicide as an antiviral immune strategy. Annu. Rev. Virol..

[185] Birkholz N., Jackson S.A., Fagerlund R.D., Fineran P.C. (2022). A mobile restriction–modification system provides phage defence and resolves an epigenetic conflict with an antagonistic endonuclease. Nucleic Acids Res..

[186] Eriksen R.S., Malhotra N., Seshasayee A.S.N., Sneppen K., Krishna S. (2022). Emergence of networks of shared restriction–modification systems in phage–bacteria ecosystems. J. Biosci..

[187] Ambroa A., Blasco L., Lopez M., Pacios O., Bleriot I., Fernandez-Garcia L., Gonzalez de Aledo M., Ortiz-Cartagena C., Millard A., Tomas M. (2021). Genomic analysis of molecular bacterial mechanisms of resistance to phage infection. Front. Microbiol..

[188] Watson B.N.J., Steens J.A., Staals R.H.J., Westra E.R., van Houte S. (2021). Coevolution between bacterial CRISPR–Cas systems and their bacteriophages. Cell Host Microbe.

[189] Liu Z., Dong H., Cui Y., Cong L., Zhang D. (2020). Application of different types of CRISPR/Cas–based systems in bacteria. Microb. Cell Fact..

[190] Nussenzweig P.M., Marraffini L.A. (2020). Molecular mechanisms of CRISPR–Cas immunity in bacteria. Annu. Rev. Genet..

[191] Ofir G., Melamed S., Sberro H., Mukamel Z., Silverman S., Yaakov G., Doron S., Sorek R. (2018). DISARM is a widespread bacterial defence system with broad anti–phage activities. Nat. Microbiol..

[192] Bravo J.P., Aparicio-Maldonado C., Nobrega F.L., Brouns S.J., Taylor D.W. (2022). Structural basis for broad anti–phage immunity by DISARM. Nat. Commun..

[193] Morgan T., Rezende R.R.d., Lima T.T.M., Souza F.d.O., Alfenas-Zerbini P. (2023). Genomic analysis unveils the pervasiveness and diversity of prophages infecting *Erwinia* species. Pathogens.

[194] Tesson F., Bernheim A. (2023). Synergy and regulation of antiphage systems: Toward the existence of a bacterial immune system?. Curr. Opin. Microbiol..

[195] Hampton H.G., Watson B.N.J., Fineran P.C. (2020). The arms race between bacteria and their phage foes. Nature.

[196] Yuan X., Huang Z., Zhu Z., Zhang J., Wu Q., Xue L., Wang J., Ding Y. (2023). Recent advances in phage defense systems and potential overcoming strategies. Biotechnol. Adv..

[197] Knecht L.E., Veljkovic M., Fieseler L. (2020). Diversity and function of phage encoded depolymerases. Front. Microbiol..

[198] Sun C.L., Barrangou R., Thomas B.C., Horvath P., Fremaux C., Banfield J.F. (2013). Phage mutations in response to CRISPR diversification in a bacterial population. Environ. Microbiol..

[199] Rostøl J.T., Marraffini L. (2019). (Ph)ighting phages: How bacteria resist their parasites. Cell Host Microbe.

[200] Kȩsik-Szeloch A., Drulis-Kawa Z., Weber-Dąbrowska B., Kassner J., Majkowska-Skrobek G., Augustyniak D., Łusiak-Szelachowska M., Zaczek M., Górski A., Kropinski A.M. (2013). Characterising the biology of novel lytic bacteriophages infecting multidrug resistant *Klebsiella pneumoniae*. Virol. J..

[201] Labrie S.J., Samson J.E., Moineau S. (2010). Bacteriophage resistance mechanisms. Nat. Rev. Microbiol..

[202] Rifat D., Wright N.T., Varney K.M., Weber D.J., Black L.W. (2008). Restriction endonuclease inhibitor IPI* of bacteriophage T4: A novel structure for a dedicated target. J. Mol. Biol..

[203] Samson J.E., Magadán A.H., Sabri M., Moineau S. (2013). Revenge of the phages: Defeating bacterial defences. Nat. Rev. Microbiol..

[204] Blower T.R., Evans T.J., Przybilski R., Fineran P.C., Salmond G.P.C. (2012). Viral evasion of a bacterial suicide system by RNA-based molecular mimicry enables infectious altruism. PLoS Genet..

[205] Połaska M., Sokołowska B. (2019). Bacteriophages—A new hope or a huge problem in the food industry. AIMS Microbiol..

[206] Goodridge L.D., Bisha B. (2011). Phage–based biocontrol strategies to reduce foodborne pathogens in foods. Bacteriophage.

[207] Abraha H.B., Kim K.P., Sbhatu D.B. (2021). Bacteriophages for detection and control of foodborne bacterial pathogens—The case of *Bacillus cereus* and their phages. J. Food Saf..

[208] Fernández L., Gutiérrez D., García P., Rodríguez A. (2019). The perfect bacteriophage for therapeutic applications—A quick guide. Antibiotics.

[209] Weber-Dąbrowska B., Jończyk-Matysiak E., Żaczek M., Łobocka M., Łusiak-Szelachowska M., Górski A.J.F. (2016). Bacteriophage procurement for therapeutic purposes. Front. Microbiol..

[210] Vázquez R., Díez-Martínez R., Domingo-Calap P., García P., Gutiérrez D., Muniesa M., Ruiz-Ruigómez M., Sanjuán R., Tomás M., Tormo-Mas M.Á. (2022). Essential topics for the regulatory consideration of phages as clinically valuable therapeutic agents: A perspective from Spain. Microorganisms.

[211] Bumunang E.W., Zaheer R., Niu D., Narvaez-Bravo C., Alexander T., McAllister T.A., Stanford K. (2023). Bacteriophages for the targeted control of foodborne pathogens. Foods.

[212] Holtappels D., Fortuna K., Lavigne R., Wagemans J. (2021). The future of phage biocontrol in integrated plant protection for sustainable crop production. Curr. Opin. Biotechnol..

[213] Farooq T., Hussain M.D., Shakeel M.T., Tariqjaveed M., Aslam M.N., Naqvi S.A.H., Amjad R., Tang Y., She X., He Z. (2022). Deploying Viruses against Phytobacteria: Potential Use of Phage Cocktails as a Multifaceted Approach to Combat Resistant Bacterial Plant Pathogens. Viruses.

[214] Frampton R.A., Pitman A.R., Fineran P.C. (2012). Advances in bacteriophage-mediated control of plant pathogens. Int. J. Microbiol..

[215] Liu S., Quek S.Y., Huang K. (2024). An Ecofriendly Nature-Inspired Microcarrier for Enhancing Delivery, Stability, and Biocidal Efficacy of Phage-Based Biopesticides. Small.

[216] Siyanbola K.F., Ejiohuo O., Ade-adekunle O.A., Adekunle F.O., Onyeaka H., Furr C.-L.L., Hodges F.E., Carvalho P., Oladipo E.K. (2024). Bacteriophages: Sustainable and Effective Solution for Climate-Resilient Agriculture. Sustain. Microbiol..

[217] Kumar P., Minnatullah M., Dhakar M., Parihar P., Choudhary M.K., Rao B.S., Mehta Y.H., Meena M., Yadav A. (2025). Harnessing Bacteriophages for Sustainable Plant Disease Management: A Review of their Potential and Market Prospects. J. Adv. Biol. Biotechnol..

[218] Yang L., Zhong W., Tang T., He M., Zhang T., Zhou B., Yin Y., Guo J., Gao Z. (2025). Phage-Based Biocontrol Strategies and Application in Aquatic Animal Disease Prevention and Control. Rev. Aquac..

[219] Jordá J., Lorenzo-Rebenaque L., Montoro-Dasi L., Marco-Fuertes A., Vega S., Marin C. (2023). Phage-Based Biosanitation Strategies for Minimizing Persistent *Salmonella* and *Campylobacter* Bacteria in Poultry. Animals.

[220] Berry E.D., Wells J.E. (2016). Reducing foodborne pathogen persistence and transmission in animal production environments: Challenges and opportunities. Microbiol. Spectr..

[221] Doyle M.P., Erickson M.C. (2012). Opportunities for mitigating pathogen contamination during on-farm food production. Int. J. Food Microbiol..

[222] Ferriol-González C., Domingo-Calap P. (2021). Phage Therapy in Livestock and Companion Animals. Antibiotics.

[223] Appiah M.O., Wang J., Lu W. (2020). Microflora in the Reproductive Tract of Cattle: A Review. Agriculture.

[224] Islam M.R., Martinez-Soto C.E., Lin J.T., Khursigara C.M., Barbut S., Anany H. (2021). A systematic review from basics to omics on bacteriophage applications in poultry production and processing. Crit. Rev. Food Sci. Nutr..

[225] Fokas R., Giormezis N., Vantarakis A. (2025). Synergistic Approaches to Foodborne Pathogen Control: A Narrative Review of Essential Oils and Bacteriophages. Foods.

[226] Abbas R.Z., Alsayeqh A.F., Aqib A.I. (2022). Role of Bacteriophages for Optimized Health and Production of Poultry. Animals.

[227] Wahab A.A.-E., Basiouni S., El-Seedi H.R., Ahmed M.F.E., Bielke L.R., Hargis B., Tellez-Isaias G., Eisenreich W., Lehnherr H., Kittler S. (2023). An overview of the use of bacteriophages in the poultry industry: Successes, challenges, and possibilities for overcoming breakdowns. Front. Microbiol..

[228] Pereira C., Duarte J., Costa P., Braz M., Almeida A. (2022). Bacteriophages in the Control of *Aeromonas* sp. in Aquaculture Systems: An Integrative View. Antibiotics.

[229] Sonne M., Ghayal N., Ahiwale S. (2023). Therapeutic application of bacteriophages against *Aeromonas* spp. mediated diseases in aquaculture: A critical review. IOSR J. Biotechnol. Biochem..

[230] Proteon Pharmaceuticals. https://www.proteonpharma.com/.

[231] Sevilla-Navarro S., Torres-Boncompte J., Garcia-Llorens J., Bernabéu-Gimeno M., Domingo-Calap P., Catalá-Gregori P. (2024). Fighting *Salmonella* Infantis: Bacteriophage-driven cleaning and disinfection strategies for broiler farms. Front. Microbiol..

[232] Sommer J., Trautner C., Witte A.K., Fister S., Schoder D., Rossmanith P., Mester P.-J. (2019). Don’t Shut the Stable Door after the Phage Has Bolted—The Importance of Bacteriophage Inactivation in Food Environments. Viruses.

[233] Tompkin R.B. (2002). Control of *Listeria monocytogenes* in the food-processing environment. J. Food Prot..

[234] Gutiérrez D., Rodríguez-Rubio L., Martínez B., Rodríguez A., García P. (2016). Bacteriophages as weapons against bacterial biofilms in the food industry. Front. Microbiol..

[235] Mgomi F.C., Yuan L., Chen C., Zhang Y., Yang Z. (2022). Bacteriophages: A weapon against mixed-species biofilms in the food processing environment. J. Appl. Microbiol..

[236] Bhandare S., Goodridge L., Harper D., Abedon S., Burrowes B., McConville M. (2021). Bacteriophages as bio-sanitizers in food production and healthcare settings. Bacteriophages.

[237] Sulakvelidze A. (2013). Using Lytic Bacteriophages to Eliminate or Significantly Reduce Contamination of Food by Foodborne Bacterial Pathogens. J. Sci. Food Agric..

[238] Chaudhary V., Kajla P., Lather D., Chaudhary N., Dangi P., Singh P., Pandiselvam R. (2024). Bacteriophages: A potential game changer in food processing industry. Crit. Rev. Biotechnol..

[239] Álvarez B., Biosca E.G. (2025). Harnessing the Activity of Lytic Bacteriophages to Foster the Sustainable Development Goals and the “One Health” Strategy. Viruses.

[240] Garvey M. (2022). Bacteriophages and Food Production: Biocontrol and Bio-Preservation Options for Food Safety. Antibiotics.

[241] Ngene A.C., Aguiyi J.C., Uzal U., Egbere J.O., Onyimba I.A., Umera A.E., Nnadi N.E. (2020). Bacteriophages as Bio-control agent against Food-Borne Pathogen *E. coli* O157: H7. IOSR J. Pharm. Biol. Sci..

[242] Wójcicki M., Świder O., Gientka I., Błażejak S., Średnicka P., Shymialevich D., Cieślak H., Wardaszka A., Emanowicz P., Sokołowska B. (2023). Effectiveness of a phage cocktail as a potential biocontrol agent against saprophytic bacteria in ready–to–eat plant–based food. Viruses.

[243] Wójcicki M., Żuwalski A.W., Świder O., Gientka I., Shymialevich D., Błażejak S. (2021). The use of bacteriophages against saprophytic mesophilic bacteria in minimally processed food. Acta Sci. Pol. Technol. Aliment..

[244] Kuek M., McLean S.K., Palombo E.A. (2023). Control of *Escherichia coli* in Fresh-Cut Mixed Vegetables Using a Combination of Bacteriophage and Carvacrol. Antibiotics.

[245] Pintor-Cora A., Carpintero A., Alegría Á., Giannis A., Lopez-Díaz T.-M., Santos J.A., Rodríguez-Calleja J.M. (2025). A Novel Bacteriophage Targeting *mcr-9 Enterobacter kobei* with Potential Application in Fresh Leafy Greens. Appl. Microbiol..

[246] Shymialevich D., Wójcicki M., Błażejak S. (2021). Wykorzystanie fagów litycznych do ograniczenia liczby pałeczek *Salmonella* w roślinnej matrycy żywnościowej [Using lytic phages to reduce the number of *Salmonella* rods in plant food matrix]. Zywn. Nauk. Technol. Ja..

[247] Wójcicki M., Swider O., Średnicka P., Shymialevich D., Ilczuk T., Koperski Ł., Cieslak H., Sokołowska B., Juszczuk-Kubiak E. (2023). Newly Isolated Virulent Salmophages for Biocontrol of Multidrug-Resistant *Salmonella* in Ready-to-Eat Plant-Based Food. Int. J. Mol. Sci..

[248] Wong C.W.Y., Wang S. (2022). Efficacy of Repeated Applications of Bacteriophages on *Salmonella enterica*-Infected Alfalfa Sprouts during Germination. Pathogens.

[249] Park E.J., Lee S., Na J.B., Kim Y.B., Lee K.M., Park S.Y., Kim J.H. (2025). Characterization of Broad Spectrum Bacteriophage vB ESM-pEJ01 and Its Antimicrobial Efficacy Against Shiga Toxin-Producing *Escherichia coli* in Green Juice. Microorganisms.

[250] Lu Y.T., Ma Y., Wong C.W., Wang S. (2022). Characterization and application of bacteriophages for the biocontrol of Shiga-toxin producing *Escherichia coli* in Romaine lettuce. Food Control.

[251] Byun K.-H., Han S.H., Choi M.W., Park S.H., Ha S.-D. (2022). Isolation, Characterization, and Application of Bacteriophages to Reduce and Inhibit *Listeria monocytogenes* in Celery and Enoki Mushroom. Food Control.

[252] Stone E., Lhomet A., Neve H., Grant I.R., Campbell K., McAuliffe O. (2020). Isolation and Characterization of *Listeria monocytogenes* Phage vB_LmoH_P61, a Phage With Biocontrol Potential on Different Food Matrices. Front. Sustain. Food Syst..

[253] Zhou C., Zhu M., Wang Y., Yang Z., Ye M., Wu L., Bao H., Pang M., Zhou Y., Wang R. (2020). Broad host range phage vB-LmoM-SH3-3 reduces the risk of *Listeria* contamination in two types of ready-to-eat food. Food Control.

[254] Wójcicki M., Średnicka P., Zapaśnik A., Emanowicz P. (2024). Risk Assessment of Foodborne and Waterborne Viruses, Detection Methods, and Possibilities of Eradication during Non-thermal Food Processing. Food Biotechnol. Agric. Sci..

[255] Raza S., Bończak B., Atamas N., Karpińska A., Ratajczyk T., Łoś M., Hołyst R., Paczesny J. (2025). The activity of indigo carmine against bacteriophages: An edible antiphage agent. Appl. Microbiol. Biotechnol..

[256] Fernández L., Escobedo S., Gutiérrez D., Portilla S., Martínez B., García P., Rodríguez A. (2017). Bacteriophages in the Dairy Environment: From Enemies to Allies. Antibiotics.

[257] Szczepankowska A.K., Górecki R.K., Kołakowski P., Bardowski J.K., Marcelino Kongo J. (2013). Lactic acid bacteria resistance to bacteriophage and prevention techniques to lower phage contamination in dairy fermentation. Lactic Acid Bacteria—R&D for Food, Health and Livestock Purposes.

[258] Pujato S., Quiberoni A., Mercanti D. (2018). Bacteriophages on dairy foods. J. Appl. Microbiol..

[259] Fox P.F., Guinee T.P., Cogan T.M., McSweeney P.L.H., Fox P.F., Guinee T.P., Cogan T.M., McSweeney P.L.H. (2017). Starter cultures. Fundamentals of Cheese Science.

[260] Zalewska-Piątek B. (2023). Phage Therapy—Challenges, Opportunities and Future Prospects. Pharmaceuticals.

[261] Szermer-Olearnik B., Boratyński J. (2015). Removal of endotoxins from bacteriophage preparations by extraction with organic solvents. PLoS ONE.

[262] Hietala V., Horsma-Heikkinen J., Carron A., Skurnik M., Kiljunen S. (2019). The removal of endo- and enterotoxins from bacteriophage preparations. Front. Microbiol..

[263] Abdelsattar A., Dawoud A., Makky S., Nofal R., Aziz R., El-Shibiny A. (2022). Bacteriophages: From isolation to application. Curr. Pharm. Biotechnol..

[264] Ali Y., Inusa I., Sanghvi G., Mandaliya V.B., Bishoyi A.K. (2023). The current status of phage therapy and its advancement towards establishing standard antimicrobials for combating multi drug-resistant bacterial pathogens. Microb. Pathog..

[265] Marks T., Sharp R. (2000). Bacteriophages and biotechnology: A review. J. Chem. Technol. Biotechnol..

[266] Choińska-Pulit A., Mituła P., Śliwka P., Łaba W., Skaradzińska A. (2015). Bacteriophage encapsulation: Trends and potential applications. Trends Food Sci. Technol..

[267] Luong T., Salabarria A.C., Edwards R.A., Roach D.R. (2020). Standardized bacteriophage purification for personalized phage therapy. Nat. Protoc..

[268] Van Belleghem J.D., Merabishvili M., Vergauwen B., Lavigne R., Mario Vaneechoutte M. (2017). A comparative study of different strategies for removal of endotoxins from bacteriophage preparations. J. Microbiol. Methods.

[269] Ali J., Rafiq Q., Ratcliffe E. (2019). A Scaled-down Model for the Translation of Bacteriophage Culture to Manufacturing Scale. Biotechnol. Bioeng..

[270] Metsemakers W.-J., Onsea J., Fintan Moriarty T., Pruidze N., Nadareishvili L., Dadiani M., Kutateladze M., Eliava G. (2023). Bacteriophage therapy for human musculoskeletal and skin/soft tissue infections. Clin. Microbiol. Infect..

[271] Liang S., Qi Y., Yu H., Sun W., Raza S.H.A., Alkhorayef N., Alkhalil S.S., Salama E.E.A., Zhang L. (2023). Bacteriophage therapy as an application for bacterial infection in China. Antibiotics.

[272] Pirnay J.-P., Verbeken G., Ceyssens P.-J., Huys I., De Vos D., Ameloot C., Fauconnier A. (2018). The magistral phage. Viruses.

[273] Djebara S., Maussen C., De Vos D., Merabishvili M., Damanet B., Pang K.W., De Leenheer P., Strachinaru I., Soentjens P., Pirnay J.-P. (2019). Processing phage therapy requests in a Brussels military hospital: Lessons identified. Viruses.

[274] Jończyk-Matysiak E., Łusiak-Szelachowska M., Kłak M., Bubak B., Międzybrodzki R., Weber-Dąbrowska B., Zaczek M., Fortuna W., Rogóz P., Letkiewicz S. (2015). The effect of bacteriophage preparations on intracellular killing of bacteria by phagocytes. J. Immunol. Res..

[275] Kawacka I., Olejnik-Schmidt A., Schmidt M., Sip A. (2020). Effectiveness of phage–based inhibition of *Listeria monocytogenes* in food products and food processing environments. Microorganisms.

[276] Fernández L., Gutiérrez D., Rodríguez A., García P. (2018). Application of bacteriophages in the agro–food sector: A long way toward approval. Front. Cell. Infect. Microbiol..

[277] Osei E.K., Mahony J., Kenny J.G. (2022). From farm to fork: *Streptococcus suis* as a model for the development of novel phage–based biocontrol agents. Viruses.

[278] Moye Z.D., Woolston J., Sulakvelidze A. (2018). Bacteriophage application for food production and processing. Viruses.

[279] Svircev A., Roach D., Castle A. (2018). Framing the future with bacteriophages in agriculture. Viruses.

[280] Jakobsen R.R., Trinh J.T., Bomholtz L., Brok-Lauridsen S.K., Sulakvelidze A., Nielsen D.S. (2022). A bacteriophage cocktail significantly reduces *Listeria monocytogenes* without deleterious impact on the commensal gut microbiota under simulated gastrointestinal conditions. Viruses.

[281] Kahn L.H., Bergeron G., Bourassa M.W., De Vegt B., Gill J., Gomes F., Malouin F., Opengart K., Ritter G.D., Singer R.S. (2019). From farm management to bacteriophage therapy: Strategies to reduce antibiotic use in animal agriculture. Ann. N. Y. Acad. Sci..

[282] Schooley R.T. (2023). Exploring bacteriophage therapy for drug–resistant bacterial infections. Top. Antivir. Med..

[283] Cobián Güemes A.G., Le T., Rojas M.I., Jacobson N.E., Villela H., McNair K., Hung S.-H., Han L., Boling L., Octavio J.C. (2023). Compounding *Achromobacter* phages for therapeutic applications. Viruses.

[284] EFSA Panel on Additives and Products or Substances used in Animal Feed (FEEDAP) (2021). Safety and efficacy of a feed additive consisting on the bacteriophages PCM F/00069, PCM F/00070, PCM F/00071 and PCM F/00097 infecting *Salmonella* Gallinarum B/00111 (Bafasal^®^) for all avian species (Proteon Pharmaceuticals SA). EFSA J..

[285] EFSA Panel on Additives and Products or Substances used in Animal Feed (FEEDAP) (2024). Safety and efficacy of a feed additive consisting of the bacteriophages PCM F/00069, PCM F/00070, PCM F/00071 and PCM F/00097 (Bafasal^®^) for all poultry (Proteon Pharmaceuticals S.A.). EFSA J..

[286] EFSA (2016). Evaluation of the safety and efficacy of Listex^TM^ P100 for reduction of pathogens on different ready–to–eat (RTE) food products. EFSA Panel on Biological Hazards. EFSA J..

[287] U.S. Food & Drug Administration GRAS Notice No. 198. https://www.cfsanappsexternal.fda.gov/scripts/fdcc/index.cfm?set=GRASNotices&id=198.

[288] U.S. Food & Drug Administration GRAS Notice No. 218. https://www.cfsanappsexternal.fda.gov/scripts/fdcc/index.cfm?set=GRASNotices&id=218.

[289] U.S. Food & Drug Administration GRAS Notice No. 528. https://www.cfsanappsexternal.fda.gov/scripts/fdcc/index.cfm?set=GRASNotices&id=528.

[290] European Medicines Agency (2022). Guideline on Quality, Safety and Efficacy of Veterinary Medicinal Products Specifically Designed for Phage Therapy.

[291] European Food Safety Authority (EFSA) (2024). EFSA statement on the requirements for whole genome sequence analysis of microorganisms intentionally used in the food chain. EFSA J..

